# Efficacy and Safety of *Lactobacillus reuteri* DSM 17938 in Pediatric Functional Abdominal Pain Disorders: A Systematic Review and Meta-Analysis of Randomized Controlled Trials

**DOI:** 10.3390/nu18172795

**Published:** 2026-08-26

**Authors:** Franco Rodríguez-Brown, Jamee Guerra Valencia, Akram Hernández-Vásquez

**Affiliations:** 1 Segunda Especialidad en Nutrición Pediátrica, Universidad Científica del Sur, Lima 15067, Peru; 100143319@cientifica.edu.pe; 2 Facultad de Ciencias de la Salud, Universidad Privada del Norte, Lima 15314, Peru; jamee.guerra@upn.pe; 3 Epidemiology and Health Economics Research (EHER), Universidad Científica del Sur, Lima 15067, Peru

**Keywords:** humans, adolescent, child, *Limosilactobacillus reuteri*, pain measurement, quality of life, abdominal pain, randomized controlled trials, systematic review, meta-analysis

## Abstract

Background/Objective: *Lactobacillus reuteri* DSM 17938 is among the most studied probiotic strains for pediatric functional abdominal pain disorders (FAPDs), but prior trials and systematic reviews have reached inconsistent conclusions. This systematic review and meta-analysis aimed to evaluate the efficacy and safety of *L. reuteri* DSM 17938 in children and adolescents with FAPDs. Methods: This review was registered in PROSPERO (CRD42024618099). PubMed, Cochrane Library, Embase, Web of Science, Scopus, CINAHL, and LILACS were searched from inception to 8 April 2026, for randomized controlled trials (RCTs) enrolling children ≤ 18 years with Rome III/IV-defined FAPDs receiving *L. reuteri* DSM 17938 versus placebo. Random-effects meta-analyses were performed for pain severity, pain frequency, quality of life, and adverse events; risk of bias was assessed with RoB 2 and certainty of evidence with GRADE. Results: Seven RCTs (513 participants) were included. Pain severity showed no significant difference at end of follow-up (SMD −0.58; 95% CI −1.32 to 0.16; I^2^ = 89.3%) but a significant reduction at end of treatment (SMD −0.49; 95% CI −0.93 to −0.06; *p* = 0.027; I^2^ = 82.0%), an effect not robust to sensitivity analysis. Pain frequency showed no significant difference at end of follow-up (MD −0.15 episodes/week; 95% CI −1.18 to 0.87) or end of treatment. No trial assessed quality of life. Adverse events were reported narratively only. Certainty of evidence was very low for pain outcomes and low for adverse events. Conclusions: Current evidence does not support a consistent beneficial effect of *L. reuteri* DSM 17938 on pain severity or frequency in pediatric FAPDs, and safety data remain limited. Certainty of evidence was low to very low across outcomes, underscoring the need for larger, methodologically rigorous trials.

## 1. Introduction

Functional abdominal pain disorders (FAPDs) comprise a spectrum of clinical conditions resulting from disturbances in gut–brain interactions and characterized by recurrent or chronic abdominal pain in the absence of an identifiable organic cause [1]. They include functional dyspepsia, irritable bowel syndrome, abdominal migraine, and functional abdominal pain—not otherwise specified [1]. Collectively, FAPDs affect approximately one in seven children and adolescents, with an estimated pooled global prevalence of 13.5%. Prevalence estimates vary according to the diagnostic criteria applied, ranging from 12.2% to 16.4%, and are further influenced by geographic region, questionnaire validation, and respondent type [2]. Their high prevalence and recurrent nature make FAPDs an important clinical problem in pediatric healthcare.

Beyond pain itself, FAPDs substantially impair children’s quality of life. Affected children experience greater school absenteeism, reduced participation in academic, recreational, and social activities, and poorer academic performance. Persistent symptoms may also increase family stress, disrupt parent–child relationships, and lead to repeated healthcare utilization [3,4]. Consequently, management should aim not only to reduce the frequency and severity of abdominal pain but also to improve daily functioning while minimizing treatment-related harms.

The pathophysiology of FAPDs is multifactorial and involves altered gastrointestinal motility, visceral hypersensitivity, impaired intestinal barrier function, immune activation, and dysregulation of bidirectional signaling along the gut–brain axis [4,5]. Alterations in the intestinal microbiota have been proposed as one of the mechanisms contributing to these processes through changes in microbial metabolite production, intestinal permeability, nociceptive signaling, and inflammatory pathways [4,6,7]. This biological rationale has stimulated growing interest in microbiota-targeted therapies, particularly probiotics.

Probiotics have been investigated as microbiota-targeted interventions for pediatric functional abdominal pain disorders. Because their biological properties and clinical effects are strain-specific, evidence should be evaluated for individual probiotic strains rather than generalized across probiotics as a whole [8]. Among these, *Lactobacillus reuteri* DSM 17938 has received particular attention. Experimental evidence suggests that it may modulate immune responses, enhance intestinal barrier integrity, and reduce visceral nociception through neuroenteric mechanisms [7]. Several randomized controlled trials have evaluated its efficacy in children with FAPDs, reporting improvements in pain intensity or pain frequency, yet results have been inconsistent [9,10,11,12,13]. Current clinical practice guidelines nevertheless address its use. The European Society for Paediatric Gastroenterology, Hepatology and Nutrition (ESPGHAN) issued a weak recommendation supporting *L. reuteri* DSM 17938 for reducing pain intensity, reflecting the low certainty of the available evidence [14].

Several systematic reviews have evaluated probiotics for pediatric functional abdominal pain disorders [8,15,16,17], and some have specifically focused on *L. reuteri* DSM 17938 [18,19,20]. Among the latter, conclusions regarding the efficacy of *L. reuteri* DSM 17938 have been inconsistent. Interpretation of the available evidence is further complicated by clinical heterogeneity across trials, including differences in diagnostic criteria, patient populations, clinical subtypes, treatment regimens, intervention duration, and outcome assessment. In addition, discrepancies among reviews may reflect methodological differences in evidence synthesis, the incorporation of newly published randomized controlled trials, or both. Consequently, uncertainty remains regarding the magnitude, durability, and clinical relevance of the treatment effect, as well as its safety profile. Therefore, we conducted a systematic review and meta-analysis of randomized controlled trials to assess the efficacy and safety of *L. reuteri* DSM 17938 compared with placebo in children and adolescents with functional abdominal pain disorders.

## 2. Materials and Methods

### 2.1. Study Design and Protocol Registration

This systematic review and meta-analysis of randomized controlled trials was conducted in accordance with a protocol registered in the International Prospective Register of Systematic Reviews (PROSPERO; registration number: CRD42024618099). The review was reported in accordance with the Preferred Reporting Items for Systematic Reviews and Meta-Analyses 2020 (PRISMA 2020) statement [21]. Methodological approaches were guided by the Cochrane Handbook for Systematic Reviews of Interventions [22].

### 2.2. Eligibility Criteria

Studies were eligible if they met the following PICOS criteria:

Population: Children and adolescents aged ≤18 years diagnosed with a pediatric functional abdominal pain disorder according to the Rome III/IV criteria.

Intervention: Administration of *L. reuteri* DSM 17938, regardless of dose, formulation, dosing frequency, or treatment duration.

Comparator: placebo.

Outcomes: Reporting of at least one prespecified efficacy or safety outcome.

Study design: Randomized controlled trials with either a parallel-group or crossover design.

Studies were excluded if the intervention combined *L. reuteri* DSM 17938 with other probiotics, prebiotics, synbiotics, or concomitant treatments that precluded isolation of the strain’s effect; if participants had abdominal pain attributable to an organic disease; or if they used a non-randomized design. Observational studies, case series, case reports, reviews, editorials, letters, conference abstracts, theses, preprints, and other non-peer-reviewed publications were also excluded. In addition, studies that did not provide sufficient data to estimate an intervention effect were excluded when the required information could not be obtained after contacting the corresponding authors. Publications in English, Spanish, and Portuguese were considered.

### 2.3. Outcomes of Interest

Outcomes were predefined in the review protocol registered in PROSPERO (CRD42024618099) and categorized as main and additional outcomes.

#### 2.3.1. Main Outcomes

Primary efficacy outcomes were: abdominal pain severity; abdominal pain episode frequency; quality of life; and adverse events.

For pain severity and pain frequency, we accepted any measurement instrument, symptom diary, or outcome definition used by the primary studies. When outcomes were reported at multiple time points, we prioritized data from the last recorded assessment for the primary analysis.

#### 2.3.2. Additional Outcomes

Additional outcomes were school absenteeism, parental or caregiver work absenteeism, additional gastrointestinal symptoms, healthcare-related costs, hospitalizations, and healthcare utilization.

### 2.4. Information Sources and Search Strategy

A systematic search was conducted in PubMed, the Cochrane Library, Embase, Web of Science Core Collection, Scopus, CINAHL, and LILACS from database inception to 8 April 2026, with no restrictions on publication date.

The initial PubMed search strategy was developed by a librarian or biomedical information specialist with experience in systematic reviews. Controlled vocabulary terms, including Medical Subject Headings (MeSH), were combined with free-text terms related to functional abdominal pain disorders, pediatric populations, and *L. reuteri* DSM 17938. Search terms were combined using Boolean operators, and the strategy was subsequently adapted to the syntax and controlled vocabularies of the other databases. The complete search strategies for all databases are provided in Supplementary Materials S1.

As supplementary search methods, the reference lists of the included studies and relevant systematic reviews were manually screened to identify additional eligible studies.

### 2.5. Study Selection

All retrieved records were initially imported into EndNote X9 (Clarivate, Philadelphia, PA, USA), where duplicates were removed using a systematic procedure based on the Bramer method [23]. The remaining records were exported in RIS format and uploaded to Rayyan to facilitate the screening process [24].

Study selection was conducted in two stages. First, two reviewers (FRB and JGV) independently screened the titles and abstracts of all retrieved records against the prespecified eligibility criteria. Second, the same reviewers independently assessed the full texts of potentially eligible studies.

Disagreements were resolved through discussion and consensus. When consensus could not be reached, a third reviewer (AHV) made the final decision. Reasons for exclusion at the full-text stage were documented and are reported in the PRISMA flow diagram.

### 2.6. Data Extraction

Two reviewers (FRB and JGV) independently extracted data in duplicate using a standardized form developed in Microsoft Excel. Before formal data extraction, the form was pilot-tested on two studies to assess the clarity of the variables and consistency between reviewers.

Discrepancies were resolved by consensus and, when necessary, through consultation with a third reviewer (AHV). Corresponding authors were contacted to request missing information or clarification of ambiguous data. When standard deviations were not reported, they were derived from standard errors, confidence intervals, *p* values, interquartile ranges, or other available summary statistics, in accordance with the recommendations of the Cochrane Handbook [22]. When outcome data were reported only in graphical format, numerical values (e.g., means, standard deviations, or other relevant summary statistics) were extracted using a digital plot digitizer (see Supplementary Materials).

### 2.7. Data Synthesis and Statistical Analysis

A narrative synthesis was conducted to summarize the methodological and clinical characteristics of the included studies, the interventions evaluated, and the reported outcomes. Clinical and methodological comparability was assessed before undertaking any quantitative synthesis.

A meta-analysis was performed when at least two studies were considered sufficiently comparable in terms of population, intervention, comparator, and outcome definition. All analyses were conducted using R version 4.5.3 (R Foundation for Statistical Computing, Vienna, Austria) with the meta package (version 8.3.0).

For continuous outcomes measured using the same scale, treatment effects were expressed as mean differences (MDs) with 95% confidence intervals (CIs). When the same outcome was measured using different instruments across studies, standardized mean differences (SMDs) were calculated as Hedges’ g and reported with 95% CIs. Whenever possible, scales were aligned so that negative values consistently indicated reduced pain or clinical improvement.

For dichotomous outcomes, including adverse events, relative risks (RRs) with 95% confidence intervals (CIs) were calculated. Count outcomes and event rates were summarized using rate ratios when studies provided sufficient information on the number of events and duration of follow-up.

When studies reported both baseline and postintervention values, change-from-baseline scores were preferred when these were available in a comparable form. When change scores were unavailable, postintervention measurements were used. Change scores and final values were combined only when permitted by the selected effect measure and when their clinical interpretation was considered equivalent.

When quantitative synthesis was possible at different assessment periods, end-of-treatment and end-of-follow-up measurements were analyzed separately. End of treatment corresponded to the assessment at completion of the intervention period, whereas end of follow-up corresponded to the final assessment after a post-treatment period without study intervention. When both assessment periods were available, the end-of-follow-up analysis was considered primary to evaluate whether treatment effects persisted after discontinuation of the intervention. Study-level assessment time points included in each quantitative synthesis are provided in Supplementary Materials S4.

Given the anticipated variability in participants’ ages, diagnostic subtypes, doses, formulations, treatment duration, and outcome measurement methods, random-effects models were used. Between-study variance (τ^2^) was estimated using the restricted maximum likelihood method. Conventional Wald-type confidence intervals were used for the primary inference. Sensitivity analyses were performed using the Hartung–Knapp–Sidik–Jonkman adjustment to account for uncertainty in the estimation of between-study heterogeneity.

Statistical heterogeneity was assessed using τ^2^, the I^2^ statistic, and Cochran’s χ^2^ test. I^2^ values were interpreted as a general guide as follows: 0–40%, heterogeneity might not be important; 30–60%, moderate heterogeneity; 50–90%, substantial heterogeneity; and 75–100%, considerable heterogeneity [22]. These thresholds were not applied rigidly; interpretation also considered the magnitude and direction of the effects and the clinical evidence of heterogeneity.

For multi-arm trials in which several relevant intervention groups shared a single placebo group, the intervention groups would be combined or the control group divided proportionally, depending on the type of outcome, to avoid unit-of-analysis errors. Eligible crossover trials would be analyzed using within-participant comparisons whenever the required data were available. If information on the within-participant correlation were insufficient, approximate analyses and sensitivity analyses would be conducted in accordance with the recommendations of the Cochrane Handbook [22].

Missing individual-level clinical outcomes were not imputed. Any imputations, estimations, or conversions based on reported summary statistics were documented and examined through sensitivity analyses.

### 2.8. Risk of Bias Assessment

The risk of bias was assessed using the revised Cochrane Risk of Bias tool for randomized trials (RoB 2) [25]. Assessments were conducted separately for each relevant outcome and reported result, considering the five RoB 2 domains. Consistent with the RoB 2 guidance, when an outcome of interest was not reported, or when the available information was insufficient to assess a specific result with RoB 2, no RoB 2 judgement was assigned for that result [25]. Instead, the implications of unavailable or incompletely reported results were considered at the review level as risk of bias due to missing results in the synthesis, in accordance with Cochrane Handbook [22]. Each domain and the overall judgement were classified as low risk of bias, some concerns, or high risk of bias, according to the RoB 2 algorithms and guidance.

Two reviewers (FRB and JGV) independently performed the risk-of-bias assessments. Disagreements were resolved through discussion and consensus or, when necessary, by consultation with a third reviewer (AHV). The results were presented in tabular and graphical formats.

### 2.9. Assessment of Reporting Bias

Reporting bias due to missing results in the synthesis was assessed following the Cochrane Handbook [22]. Outcomes described in the Methods sections of the trial reports, trial registry entries, or available study protocols were compared with the published results to identify prespecified outcomes for which study results were unavailable for inclusion in the review. A reporting matrix was constructed for each outcome to document whether study results were available for inclusion in the synthesis, unavailable because the outcome was probably not assessed, or unavailable despite the outcome having been considered in the study. In the latter case, the possibility that results were missing because of their *p* value, magnitude, or direction was assessed following the criteria described in the Cochrane Handbook. Briefly, this approach considers it reasonable to suspect such bias when there is evidence that an outcome was measured but no results are available without explanation, or when only incomplete information, such as a narrative statement regarding the direction or statistical significance of the result, is reported. This matrix informed the assessment of risk of bias due to missing results in the synthesis.

Assessment of small-study effects using funnel plots and appropriate statistical tests for funnel plot asymmetry was prespecified for meta-analyses including at least 10 studies [26]. However, as all meta-analyses included fewer than 10 studies, these assessments were not performed.

Outcomes prespecified in the methods sections of the included articles, trial registries, or available protocols were compared with the published results to identify outcomes that may have been measured but not reported.

### 2.10. Certainty of Evidence Assessment

The certainty of the evidence for each primary outcome was assessed using the Grading of Recommendations Assessment, Development and Evaluation (GRADE) approach. Because randomized controlled trials were included, the certainty of evidence was initially rated as high and was downgraded, when appropriate, for risk of bias, inconsistency, indirectness, imprecision, and publication bias. The certainty of evidence for each outcome was classified as high, moderate, low, or very low.

Imprecision was assessed following the GRADE guidance for minimally contextualized interpretation, considering the width of the confidence intervals, whether they crossed predefined thresholds of clinical importance, and whether the optimal information size (OIS) was achieved [27].

Results were summarized in Summary of Findings (SoF) tables prepared using the web-based version of GRADEpro GDT (GRADE Working Group, Hamilton, ON, Canada). The SoF tables included relative and absolute effect estimates, the number of participants and studies, the certainty of the evidence, and explanations for all downgrading decisions. Detailed GRADE evidence profiles are provided in the Supplementary Materials.

### 2.11. Ethical Considerations

This review used exclusively data from previously published studies and was conducted as part of a broader research project academically approved by the Second Specialty Program in Pediatric Nutrition at Universidad Científica del Sur (Protocol No. POS-119-2024-01072). In accordance with the University’s regulations (Resolución Directoral No. 008-DGIDI-CIENTIFICA-2023), research projects based exclusively on the analysis of previously published studies are exempt from review by the Institutional Research Ethics Committee.

## 3. Results

### 3.1. Search Results

**Figure 1:**
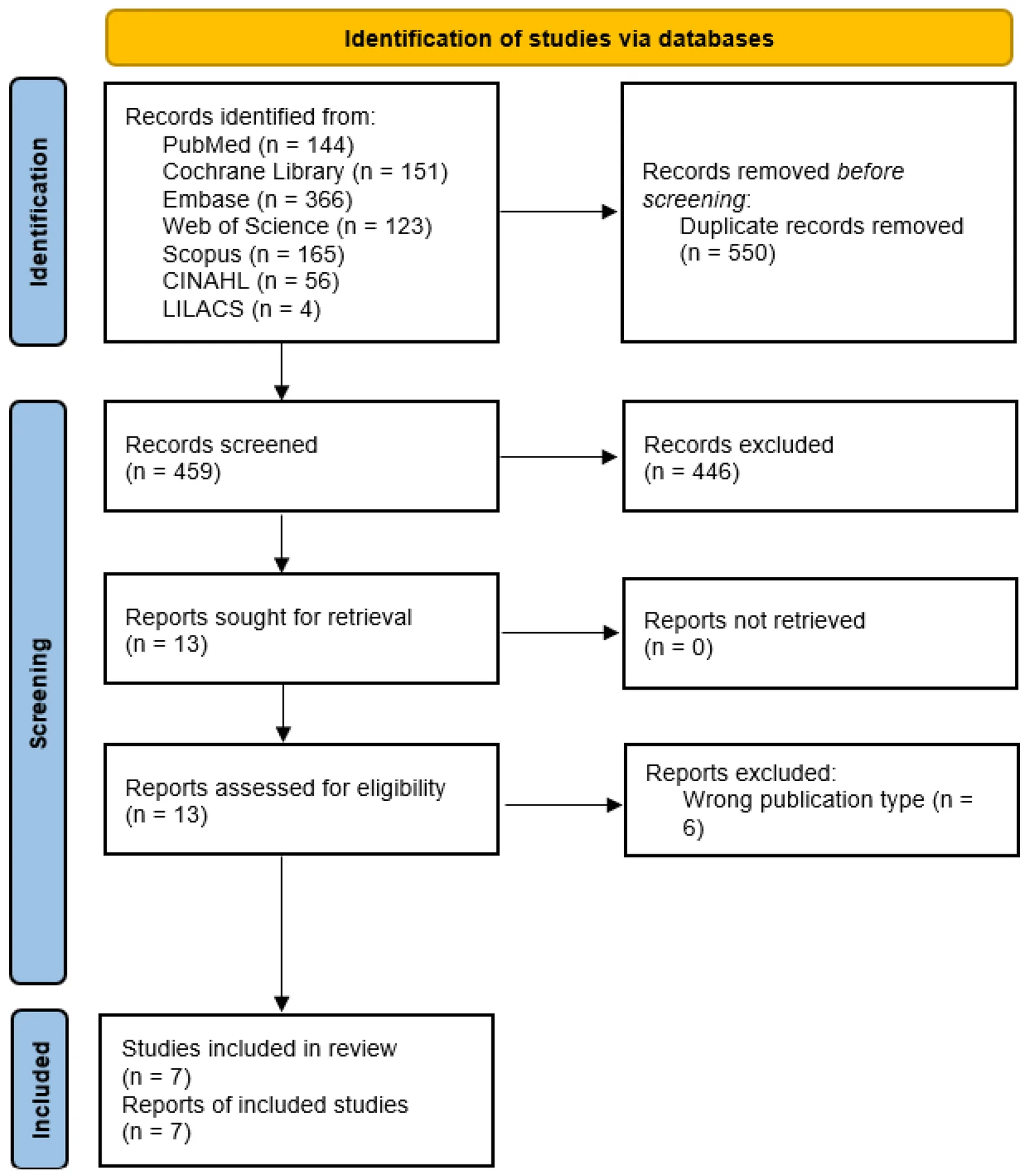
PRISMA 2020 flow diagram of the study selection process.

After duplicate elimination we obtained a total of 459 records, which were screened by title and abstract (Figure 1). A total of 13 reports were assessed for eligibility and 7 RCTs were included [9,10,11,12,13,28,29]. Excluded studies and reasons for exclusion are presented in Supplementary Materials.

### 3.2. Study Characteristics

A total of seven parallel-group randomized controlled trials involving 513 participants were included in the review [9,10,11,12,13,28,29]. The studies were published between 2014 and 2020 and were conducted across seven countries, including Italy, Iran, Israel, Greece, Slovenia, Poland, and Croatia. Participants were children and adolescents with functional abdominal pain disorders, primarily diagnosed according to the Rome III criteria. The included populations comprised children with functional abdominal pain, irritable bowel syndrome, functional dyspepsia, abdominal migraine, or mixed functional abdominal pain disorders.

All studies evaluated oral supplementation with *L. reuteri* DSM 17938 compared with placebo. The probiotic was administered as liquid drops or chewable tablets at daily doses of 1 × 10^8^ or 2 × 10^8^ colony-forming units (CFU). The intervention period ranged from 4 to 12 weeks. Six studies also included an additional follow-up period after discontinuation of the intervention, during which participants no longer received *L. reuteri* or placebo. This post-intervention follow-up lasted four weeks in all six studies [9,10,11,12,13,28]. Sample sizes ranged from 46 to 125 participants. Pain severity and pain frequency were the most commonly reported outcomes, whereas quality of life, adverse events, school absenteeism, parental or caregiver work absenteeism, additional gastrointestinal symptoms, healthcare-related costs, hospitalizations, and healthcare utilization were reported less consistently across studies (Table 1).

### 3.3. Risk of Bias in Studies

**Figure 2:**
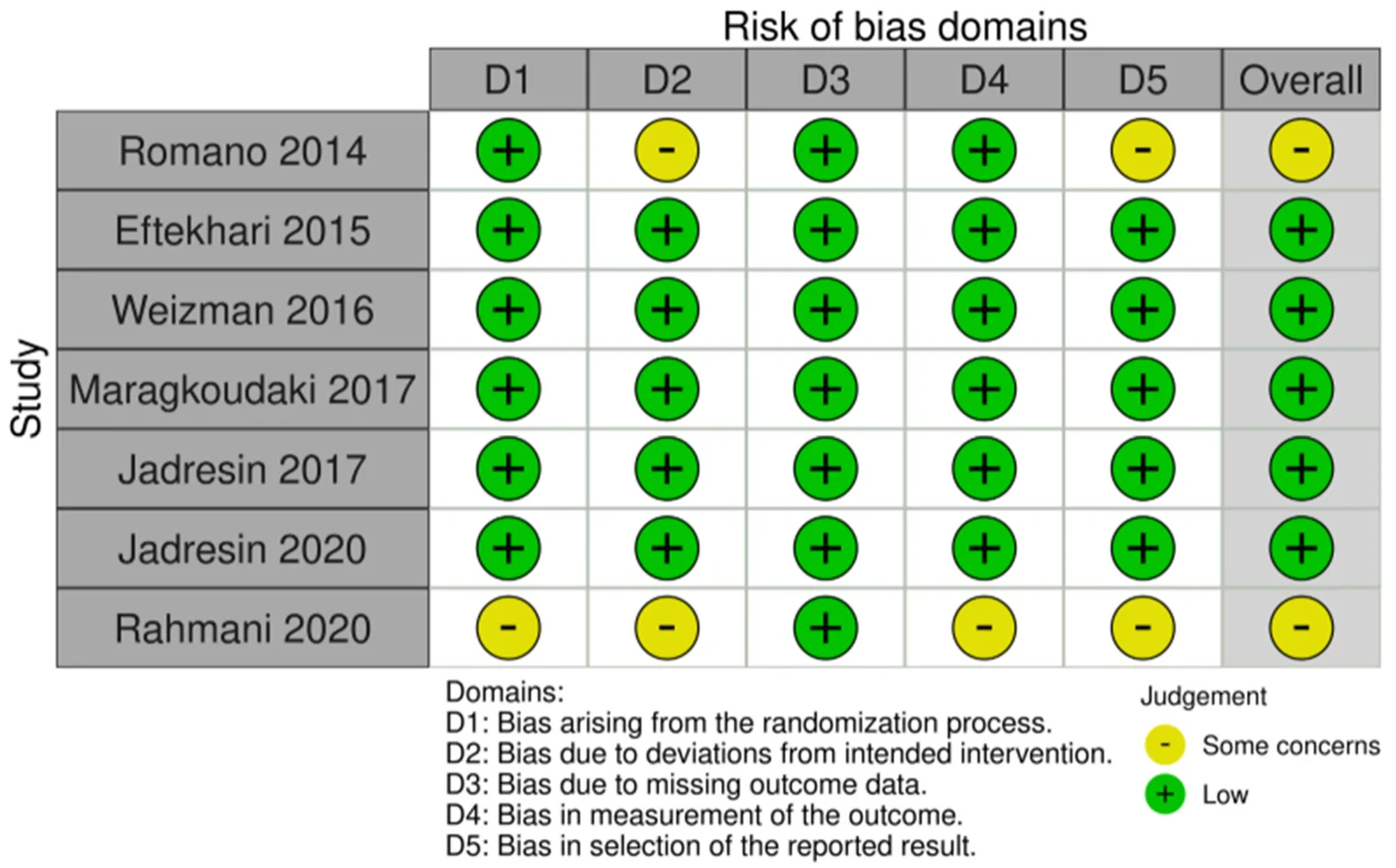
Risk of bias assessment across Cochrane domains for pain severity. Studies included: Romano et al. [9], Eftekhari et al. [28], Weizman et al. [10], Maragkoudaki et al. [12], Jadresin et al. [11,13], Rahmani et al. [29].

**Figure 3:**
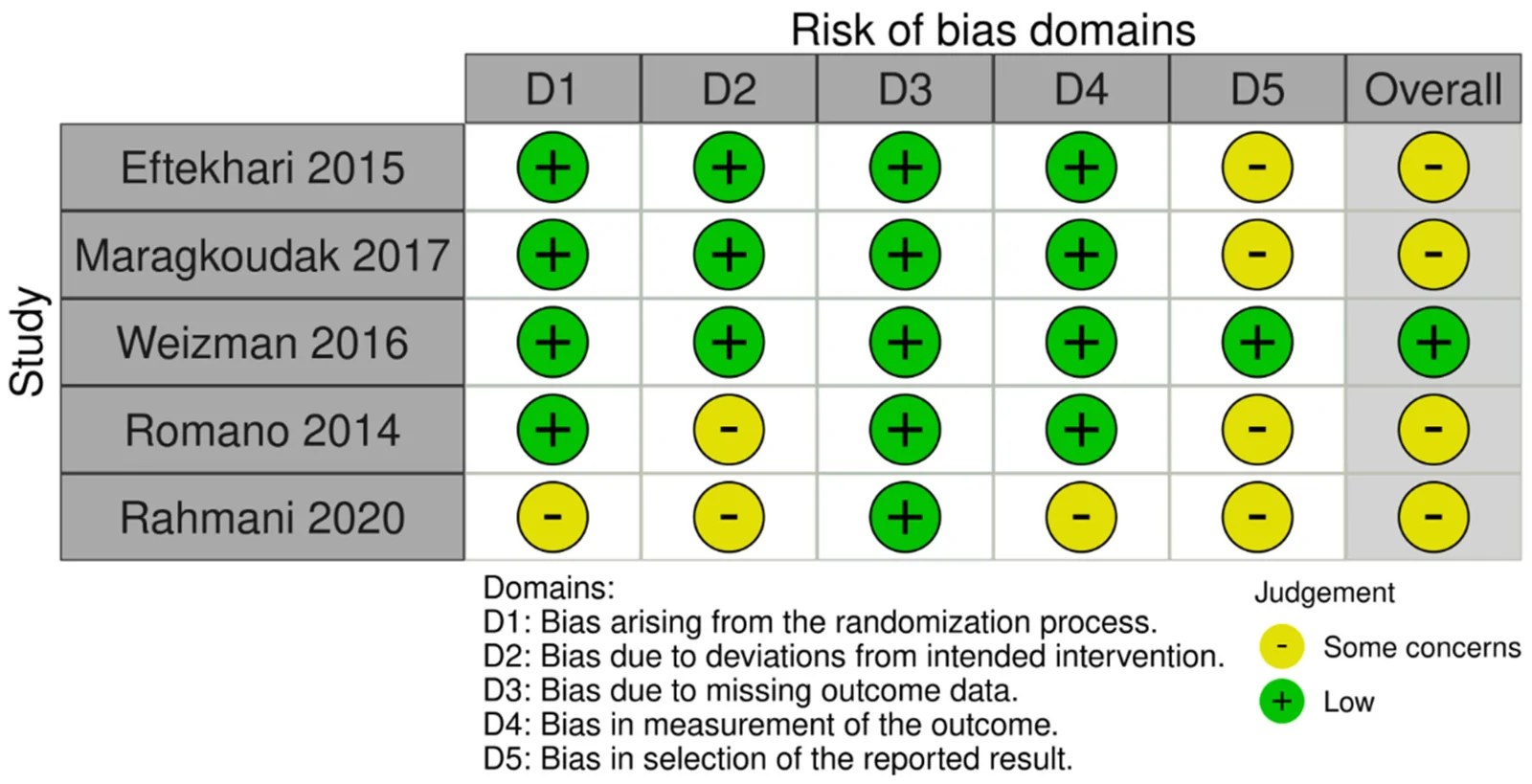
Risk of bias assessment across Cochrane domains for pain frequency. Studies included: Eftekhari et al. [28], Maragkoudaki et al. [12], Weizman et al. [10], Romano et al. [9], Rahmani et al. [29].

**Figure 4:**
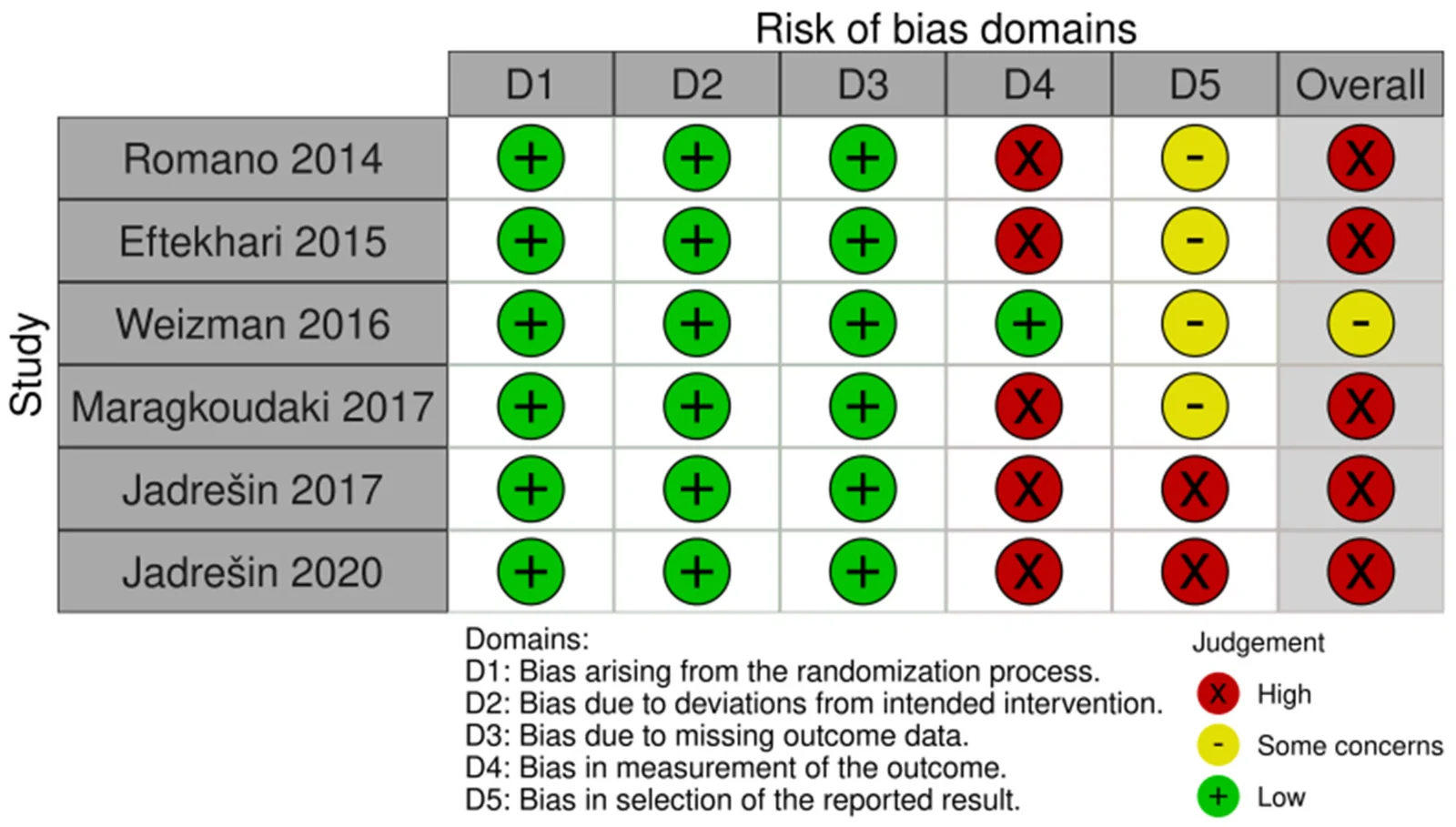
Risk of bias assessment across Cochrane domains for adverse events. Studies included: Romano et al. [9], Eftekhari et al. [28], Weizman et al. [10], Maragkoudaki et al. [12], Jadresin et al. [11,13].

Risk of bias was assessed separately for each outcome using the RoB 2 tool. Traffic-light plots for the primary outcomes, including pain intensity (7 RCT), pain frequency (5 RCT), and adverse events (6 RCT), are presented in Figure 2, Figure 3 and Figure 4. The detailed domain-level judgements and supporting explanations for all outcomes, are provided in the Supplementary Materials.

For the pain outcomes (severity and frequency), the randomization process (D1) and missing outcome data (D3) were generally judged as low risk of bias. Outcome measurement (D4) also showed a predominantly low risk profile, whereas some concerns in pain intensity were additionally identified in deviations from intended interventions (D2). For pain frequency, D5 represented the main source of concern because insufficient information was available to determine whether the reported outcome measurements and analyses had been prespecified. In contrast, adverse events showed a less favorable risk-of-bias profile. Although D1, D2, and D3 were consistently judged as low risk, most studies were rated at high risk in D4 because adverse events were neither operationally defined nor assessed using clearly described ascertainment procedures. Concerns regarding selection of the reported result were also common because adverse events were frequently not prespecified in trial protocols or registrations.

Among the additional outcomes, RoB 2 assessments for school absenteeism, additional gastrointestinal symptom frequency and severity, and medication use included only studies reporting outcome-specific results. The implications of unavailable or incompletely reported results from eligible studies were evaluated separately as risk of bias due to missing results in the synthesis and are presented in the Reporting Bias section. In contrast, the only study reporting parental or caregiver work absenteeism was judged as having a low risk of bias across all RoB 2 domains.

### 3.4. Main Outcomes: Pain, Quality of Life, and Adverse Events

**Figure 5:**
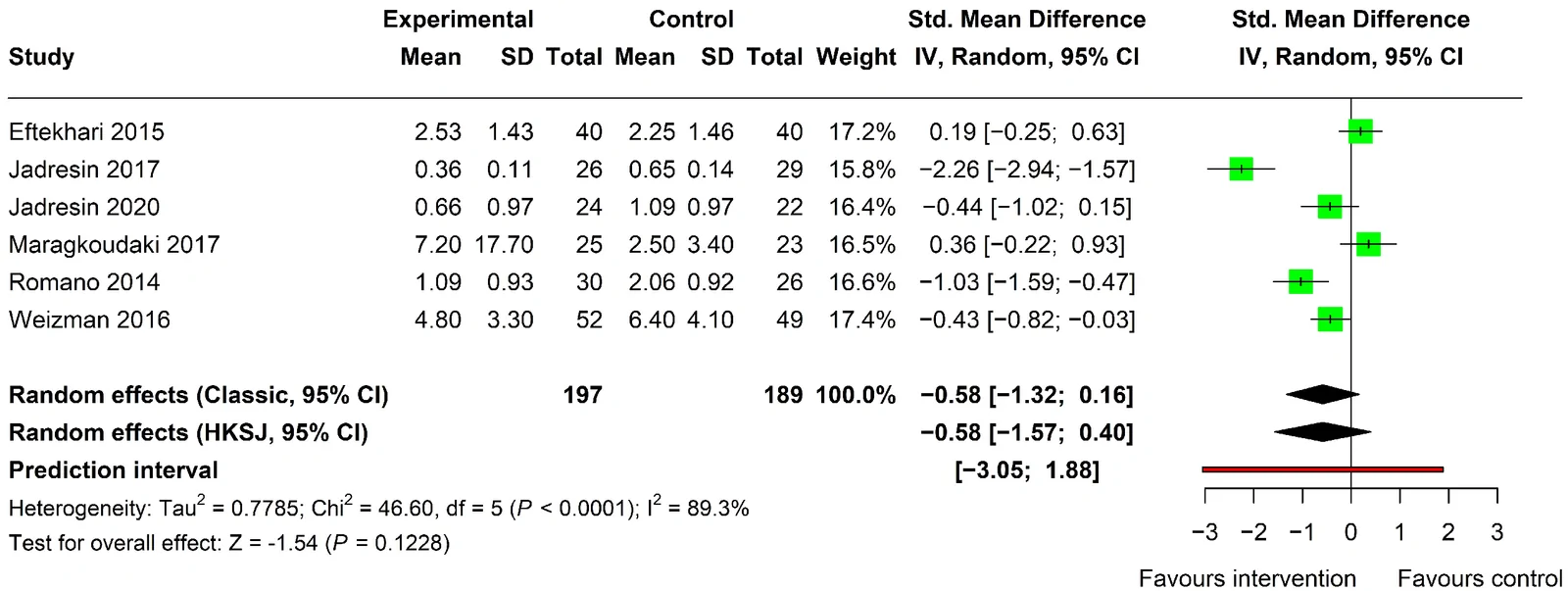
Forest plots of the effects of pain intensity based on end-of-follow-up measurements [9,10,11,12,13,28]. Green squares represent individual study effect estimates, with the center indicating the point estimate and the size proportional to study weight. Horizontal lines indicate 95% confidence intervals. Black diamonds represent pooled estimates from random-effects models with between-study variance (τ^2^) estimated using REML. The upper diamond uses conventional inference and the lower diamond uses the Hartung–Knapp–Sidik–Jonkman adjustment, with the center indicating the pooled estimate and the width representing the 95% confidence interval. The vertical solid line represents the line of no effect (SMD = 0). CI, confidence interval; I^2^, heterogeneity; Tau^2^, between-study variance.

**Figure 6:**
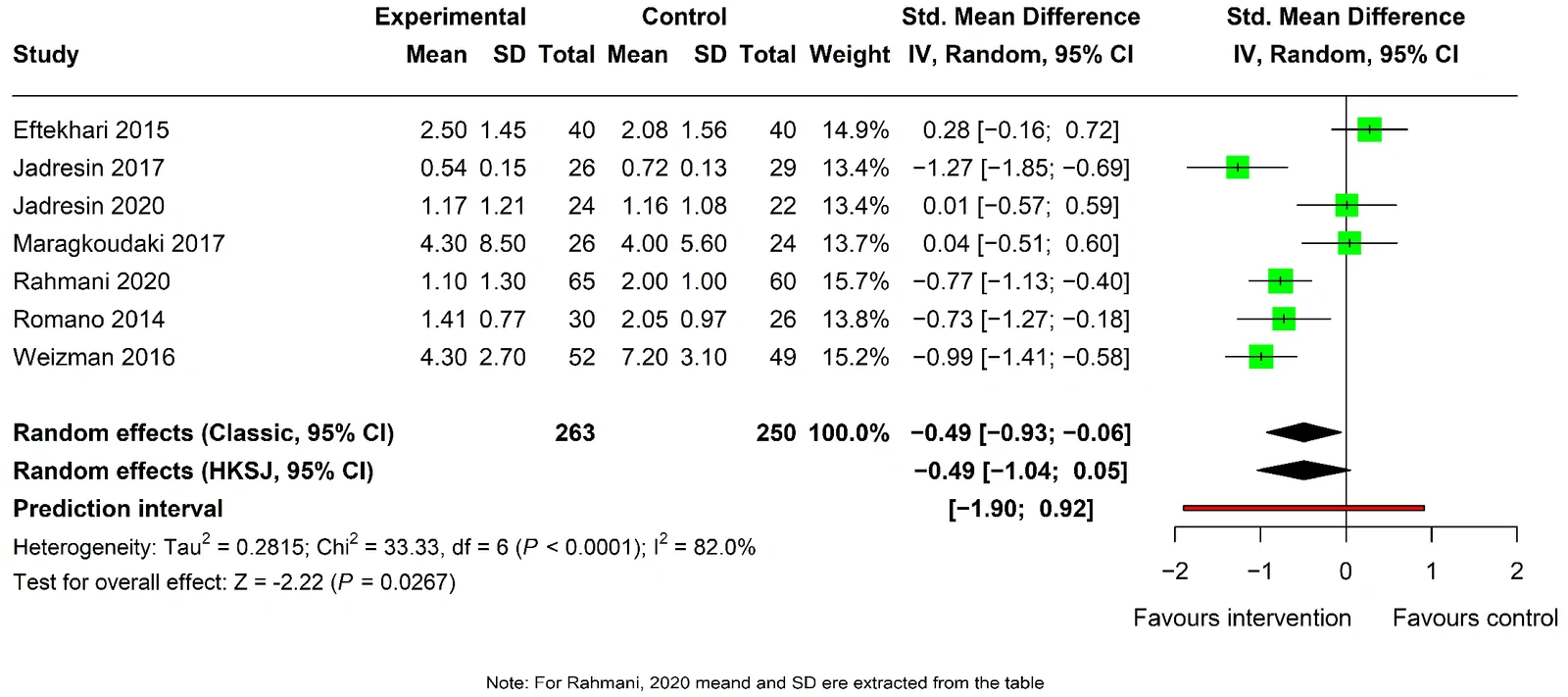
Forest plots of the effects of pain intensity based on end-of-treatment measurements [9,10,11,12,13,28,29]. Green squares represent individual study effect estimates, with the center indicating the point estimate and the size proportional to study weight. Horizontal lines indicate 95% confidence intervals. Black diamonds represent pooled estimates from random-effects models with between-study variance (τ^2^) estimated using REML. The upper diamond uses conventional inference and the lower diamond uses the Hartung–Knapp–Sidik–Jonkman adjustment, with the center indicating the pooled estimate and the width representing the 95% confidence interval. The vertical solid line represents the line of no effect (SMD = 0). CI, confidence interval; I^2^, heterogeneity; Tau^2^, between-study variance.

**Figure 7:**
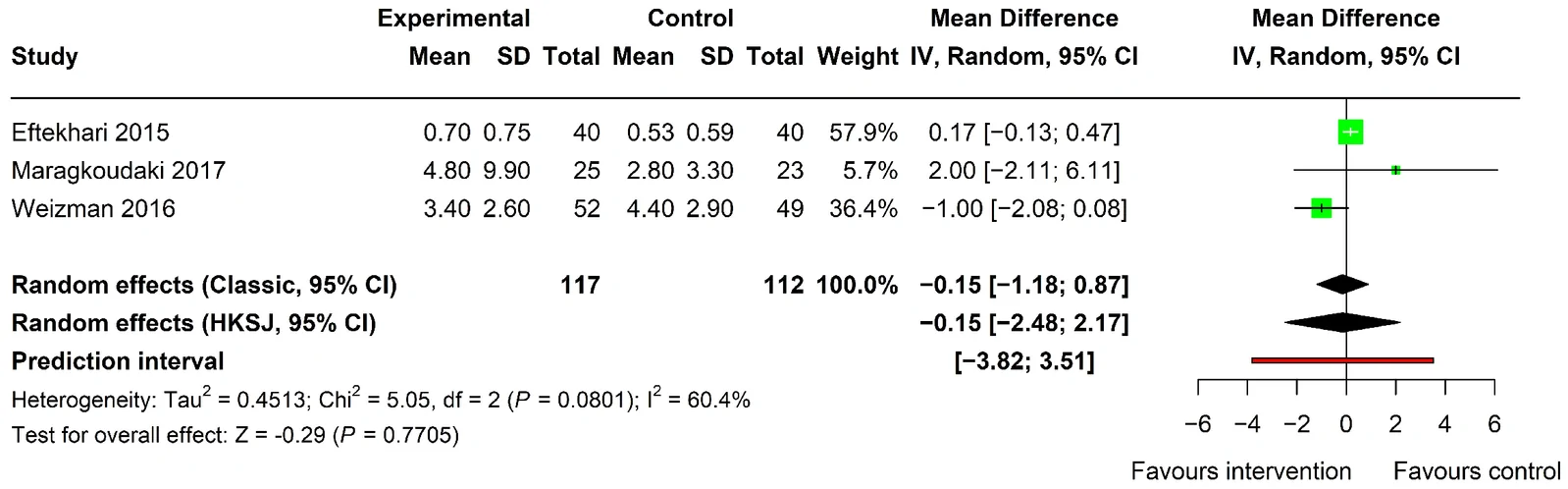
Forest plots of the effects of pain frequency based on end-of-follow-up measurements [10,12,28]. Green squares represent individual study effect estimates, with the center indicating the point estimate and the size proportional to study weight. Horizontal lines indicate 95% confidence intervals. Black diamonds represent pooled estimates from random-effects models with between-study variance (τ^2^) estimated using REML. The upper diamond uses conventional inference and the lower uses the Hartung–Knapp–Sidik–Jonkman adjustment, with the center indicating the pooled estimate and the width representing the 95% confidence interval. The vertical solid line represents the line of no effect (MD = 0). CI, confidence interval; I^2^, heterogeneity; Tau^2^, between-study variance.

**Figure 8:**
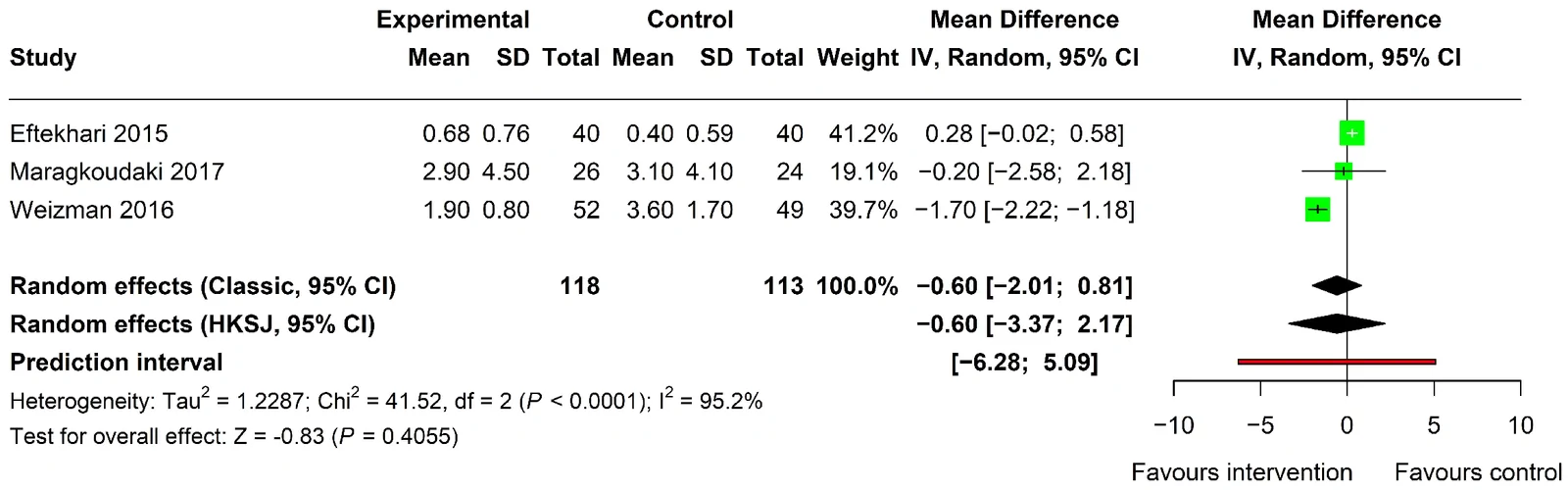
Forest plots of the effects of pain frequency based on end-of-treatment measurements [10,12,28]. Green squares represent individual study effect estimates, with the center indicating the point estimate and the size proportional to study weight. Horizontal lines indicate 95% confidence intervals. Black diamonds represent pooled estimates from random-effects models with between-study variance (τ^2^) estimated using REML. The upper diamond uses conventional inference and the lower uses the Hartung–Knapp–Sidik–Jonkman adjustment, with the center indicating the pooled estimate and the width representing the 95% confidence interval. The vertical solid line represents the line of no effect (MD = 0). CI, confidence interval; I^2^, heterogeneity; Tau^2^, between-study variance.

The primary outcomes comprised pain severity, pain frequency, quality of life, and adverse events. An overview of the evidence available for each outcome is presented in Table 2. Forest plots for the quantitative syntheses of pain severity and pain frequency are presented in Figure 5, Figure 6, Figure 7 and Figure 8. Prespecified subgroup and sensitivity analyses are provided in the Supplementary Materials.

*Pain severity*

Seven RCTs evaluated pain severity [9,10,11,12,13,28,29]. Six RCTs, comprising 386 participants, contributed to the primary meta-analysis, which was based on end-of-follow-up measurements [9,10,11,12,13,28], with the study by Margkoudaki et al. [12] including one fewer participant in each treatment arm at follow-up. All seven RCTs, comprising 513 participants, contributed to a secondary meta-analysis based on end-of-treatment measurements (Figure 5 and Figure 6). The primary analysis showed no statistically significant difference between *L. reuteri* DSM 17938 and the control group (SMD: −0.58; 95% CI: −1.32 to 0.16; *p* = 0.123; I^2^ = 89.3%). In contrast, the secondary analysis based on end-of-treatment measurements showed a statistically significant reduction in pain severity favoring *L. reuteri* DSM 17938 (SMD: −0.49; 95% CI: −0.93 to −0.06; *p* = 0.027; I^2^ = 82.0%). However, this result was not robust to the HKSJ adjustment, for which the 95% CI included the null (SMD: −0.49; 95% CI: −1.04 to 0.05).

A prespecified sensitivity analysis using the alternative end-of-treatment values reported in the text rather than the corresponding table for Rahmani et al., 2020 [29], yielded a non-statistically significant pooled effect (SMD: −0.34; 95% CI: −0.81 to 0.12; *p* = 0.147; I^2^ = 85.0%) (Supplementary Figure S1). Prespecified subgroup analyses according to follow-up duration, supplement formulation, and probiotic dose did not provide evidence of subgroup differences (Supplementary Figures S2–S4).

*Pain frequency*

Five RCTs evaluated pain frequency [9,10,12,28,29]. Three contributed to the quantitative synthesis because they reported the outcome as the number of episodes per week [10,12,28], whereas two studies reported pain episodes using different or unspecified time units and were therefore not included in the meta-analysis (Figure 7 and Figure 8). In particular, Romano 2014 reported the outcome as episodes/day [9] and showed no statistically significant difference between groups (estimated MD: 0.08 episodes/day; 95% CI: −0.36 to 0.52), whereas Rahmani 2020 reported the number of pain episodes without specifying the time unit [29] and found a statistically significant reduction favoring *L. reuteri* DSM 17938 (estimated MD: −2.7; 95% CI: −4.16 to −1.24).

Among the three studies included in the meta-analysis [10,12,28], the primary analysis based on end-of-follow-up measurements showed no statistically significant difference between *L. reuteri* DSM 17938 and the control group (MD: −0.15 episodes/week; 95% CI: −1.18 to 0.87; *p* = 0.771; I^2^ = 60.4%). The secondary meta-analysis based on end-of-treatment measurements also showed no statistically significant effect (MD: −0.60 episodes/week; 95% CI: −2.01 to 0.81; *p* = 0.406; I^2^ = 95.2%). Prespecified subgroup analyses of the primary end-of-follow-up meta-analysis, according to supplement formulation and probiotic dose, did not provide evidence of subgroup differences (Supplementary Figures S5 and S6).

*Quality of life*

None of the included RCTs evaluated or reported quality of life. Consequently, no evidence was available for this outcome.

*Adverse events*

Six RCTs evaluated adverse events [9,10,11,12,13,28]. All studies reported the outcome narratively; therefore, quantitative synthesis was not possible. Four studies reported no adverse events in either study group [9,10,11,13], whereas two studies reported no adverse events among participants receiving *L. reuteri* DSM 17938 [12,28]. Methods of adverse-event surveillance or ascertainment and specific observation periods for adverse events were not consistently reported across trials.

*Additional outcomes*

Five additional outcomes were identified: school absenteeism, parental or caregiver work absenteeism, additional gastrointestinal symptoms (frequency and severity), and healthcare utilization assessed as rescue medication use.

School absenteeism was assessed in five RCTs; however, substantial variability in outcome reporting precluded quantitative synthesis. Two studies provided quantitative estimates. For Maragkoudaki et al., 2017 [12], there was no statistically significant difference between groups (estimated MD: −0.11 days; 95% CI: −0.32 to 0.10), whereas for Weizman et al., 2016 [10] fewer days of school absenteeism in the *L. reuteri* DSM 17938 group was found (estimated MD: −0.80 days; 95% CI: −1.20 to −0.40). Two trials (Jadrešin et al., 2017; Jadrešin et al., 2020) reported the outcome narratively as exploratory [11,13], indicating no between-group differences, and Rahmani et al., 2020 included school absenteeism within a broader outcome named “disturbance activities” but did not provide outcome-specific quantitative results for school absenteeism [29].

Parental or caregiver work absenteeism was reported by a single RCT [12]. No statistically significant difference between groups was found (estimated MD: −0.12 days/week; 95% CI: −0.35 to 0.11).

Four RCTs evaluated additional gastrointestinal symptoms. Quantitative data on symptom frequency were available from two studies only. Weizman et al., 2016 [10] reported fewer episodes per week in the *L. reuteri* DSM 17938 group (estimated MD: −1.50 episodes/week; 95% CI: −2.10 to −0.90), whereas Maragkoudaki et al., 2017 [12] reported a lower frequency of nausea at the mid-treatment assessment (estimated Risk Difference: −0.29; 95% CI: −0.54 to −0.04). For symptom severity, only Maragkoudaki et al., 2017 [12] provided quantitative data, showing lower gastrointestinal symptom scores in the intervention group (estimated MD: −7.10; 95% CI: −13.85 to −0.35). Romano et al., 2014 and Eftekhari et al., 2015 [9,28] prespecified these outcomes but reported only narrative statements, indicating no differences or no unexpected gastrointestinal symptoms, whereas Weizman et al., 2016 [10] qualitatively reported no differences in symptoms other than abdominal distension and bloating.

Healthcare utilization, assessed as rescue medication use, was evaluated in five RCTs [9,10,12,28,29]. Two studies reported quantitative data at the end of treatment [12,29]; however, one trial reported a standard deviation of zero [29], precluding quantitative synthesis without imputation. Consequently, the analysis without imputation included only Maragkoudaki et al., 2017 [12] and showed no statistically significant difference between groups (MD: 0.07 days/week; 95% CI: −0.07 to 0.21; *p* = 0.318). To incorporate both studies, the primary quantitative synthesis used the SD observed in the control group of Maragkoudaki et al., 2017 [12] (SD = 0.07) for the Rahmani et al., 2020 [29] trial. The pooled estimate remained non-statistically significant (MD: 0.03 days/week; 95% CI: −0.01 to 0.08; *p* = 0.157; I^2^ = 0%). Sensitivity analyses using a reduced borrowed SD (SD = 0.035) and a near-zero SD (SD = 0.0001) yielded virtually identical pooled estimates and did not alter the overall conclusion. The primary and sensitivity meta-analyses are presented in Supplementary Materials (Supplementary Figures S7–S9 and Supplementary Table S1). Romano et al., 2014 and Eftekhari et al., 2015 prespecified rescue medication use but did not report results [9,28], whereas Weizman et al., 2016 reported medication use for abdominal pain only as a baseline characteristic [10].

### 3.5. Reporting Bias

Formal assessment of publication bias using funnel plots or statistical tests for funnel plot asymmetry was not performed because all meta-analyses included fewer than 10 studies.

The availability of outcome results across included studies is summarized in Table 3. Potential risk of bias due to missing results in the synthesis was identified for school absenteeism, additional gastrointestinal symptoms (frequency and severity), and healthcare utilization (rescue medication use). Across these outcomes, several studies prespecified the outcomes in their methods sections or trial registrations but did not report quantitative results, reported only narrative findings, or reported broader composite outcomes that precluded extraction of outcome-specific estimates. Consequently, the possibility that results were unavailable because of their direction, magnitude, or statistical significance could not be excluded.

In contrast, no important concerns regarding missing results in the synthesis were identified for the primary outcomes of pain severity, pain frequency, quality of life, or adverse events.

### 3.6. Certainty of Evidence

The certainty of the evidence for all outcomes is summarized in the Summary of Findings tables (Table 4 and Table 5), with the corresponding GRADE evidence profiles provided in the Supplementary Materials.

For the primary outcomes, the certainty of the evidence was rated as very low for pain severity at the end of follow-up, pain severity at the end of treatment, pain frequency at the end of follow-up, and pain frequency at the end of treatment. The certainty of the evidence for adverse events was low (Table 4).

Among the additional outcomes, the certainty of the evidence was very low for school absenteeism, additional gastrointestinal symptoms (frequency and severity), and healthcare utilization (rescue medication use), whereas low-certainty evidence was identified for parental or caregiver work absenteeism (Table 5).

## 4. Discussion

The aim of this systematic review and meta-analysis was to evaluate the effects of *L. reuteri* DSM 17938 for the treatment of functional abdominal pain disorders in children. Overall, a statistically significant reduction in pain severity was observed at the end of treatment but not at the end of follow-up, which was considered the primary analysis. Likewise, no statistically significant reduction was observed in pain frequency at either the end of treatment or the end of follow-up. No included trial evaluated quality of life, and adverse events were infrequently reported, with all studies describing either no adverse events or no adverse events among participants receiving *L. reuteri* DSM 17938. Evidence for the additional outcomes was limited and heterogeneous. Quantitative synthesis was not possible for most outcomes because of differences in outcome definitions, measurement methods, time points, or incomplete reporting across studies. The certainty of the evidence ranged from low to very low across outcomes. Overall, confidence in the estimated effects was limited because of concerns regarding risk of bias, inconsistency, imprecision, and, for some outcomes, suspected publication bias.

Previous systematic reviews evaluating *L. reuteri* DSM 17938 for pediatric FAPDs have reached differing conclusions [8,18,19,20,30]. The earliest reviews, including the systematic review by Wegh et al., 2018 [18], and the rapid review by Ding et al., 2019 [20], synthesized evidence from the same five randomized controlled trials included in the present review [9,10,11,12,28] and concluded that the available evidence was insufficient or inconsistent to support the routine use of *L. reuteri* DSM 17938. These conclusions are consistent with the overall interpretation of our findings despite the incorporation of two additional randomized trials. In contrast, the subsequent systematic review and meta-analysis by Trivić et al., 2021 [19] which included six of the seven trials incorporated in our review [9,10,11,12,13,28], concluded that *L. reuteri* DSM 17938 reduced pain intensity and increased the number of pain-free days in children with FAPDs. Notably, despite these favorable conclusions, the certainty of the evidence for pain intensity was rated as very low at both 8- and 12-week follow-up assessments and low at 4 weeks.

An important methodological difference between previous reviews and the present study concerns the quantitative synthesis strategy. For pain severity, Trivić et al., 2021 pooled continuous outcomes using mean differences [19] despite one of the included trials [10] assessing pain with the Faces Pain Scale-Revised, whereas the remaining trials used the Wong-Baker FACES Pain Rating Scale. In the present review, standardized mean differences were used to account for these differences in measurement instruments. Likewise, for pain frequency, Trivić et al., 2021 [19] quantitatively combined Romano et al., 2014, which reported the outcome as episodes/day, with studies reporting episodes/week [9]. In contrast, our meta-analysis was restricted to studies reporting comparable outcome definitions and units of measurement. These methodological differences may influence the pooled estimates and should therefore be considered when interpreting differences between reviews. Furthermore, the 2022 ESPGHAN position paper [30], based its recommendation for *L. reuteri* DSM 17938 largely on the evidence synthesized by Wegh et al., 2018 and Trivić et al., 2021 [18,19]. Although the guideline acknowledged the insufficient evidence identified by Wegh et al., 2018 [18], it highlighted the statistically significant reduction in pain intensity reported by Trivić et al., 2021 [19] and consequently issued a weak recommendation, supported by moderate certainty of evidence, for the use of *L. reuteri* DSM 17938 to reduce pain intensity in children with FAPDs [30]. More recently, Yang et al. performed a network meta-analysis including all randomized trials incorporated in the present review and reported statistically significant reductions in both pain severity and pain frequency with *L. reuteri* DSM 17938 [8]. However, the methods used to account for differences in pain measurement scales for pain severity and differences in the reporting time units for pain frequency were not fully described, making it difficult to determine how these sources of clinical and methodological heterogeneity were addressed before quantitative synthesis. Consequently, direct comparison between those findings and the present review should be made with caution. Taken together, the primary evidence base has changed only marginally over time, whereas the conclusions of successive evidence syntheses have evolved. This pattern suggests that differences between reviews are unlikely to be explained solely by the availability of new randomized trials, but also by differences in the quantitative synthesis strategies applied to a relatively small and heterogeneous body of evidence. In addition, unlike previous reviews, the present study explicitly evaluated missing results in the synthesis according to Cochrane guidance and incorporated these assessments into both the evaluation of reporting bias and the GRADE certainty ratings, thereby providing a more comprehensive appraisal of the certainty and interpretability of the available evidence.

The discrepancy between the end-of-treatment and end-of-follow-up findings for pain severity deserves careful consideration. Although a statistically significant reduction in pain severity was observed at the end of treatment, this effect was not maintained at the end of follow-up, which was considered the primary analysis to assess whether any treatment effect persisted after discontinuation of the probiotic. The apparent short-term benefit should therefore be interpreted cautiously. The end-of-treatment meta-analysis incorporated one additional trial [29], increasing the available sample size by approximately 125 participants. However, this finding was not robust, as the HKSJ adjustment yielded a confidence interval that included the null, and statistical significance was lost in the prespecified sensitivity analysis using the alternative values reported in the study. Moreover, subgroup analyses based on the primary end-of-follow-up analysis did not identify differences according to follow-up duration, supplement formulation, or probiotic dose, but the small number of trials limited the exploration of potential sources of heterogeneity. The magnitude of the end-of-treatment pooled estimate (SMD: −0.49) would conventionally be considered moderate. However, its confidence interval encompassed effects ranging from trivial to large, and substantial between-study heterogeneity was accompanied by a wide prediction interval spanning effects in both directions. Potential sources of heterogeneity included differences in FAPD subtypes, participant characteristics, treatment and follow-up duration, probiotic dose and formulation, and pain assessment methods. Although some of these factors were explored in subgroup analyses, the small number of trials limited our ability to identify specific sources of heterogeneity. In the absence of an established minimally important difference for pain severity in children with FAPDs, the clinical importance of this magnitude remains uncertain. Taken together, these findings suggest that the current evidence does not support a sustained reduction in pain severity with *L. reuteri* DSM 17938. This interpretation is consistent with the very low certainty of the evidence, indicating that the true effect may be substantially different from the estimated effect. Similarly, no statistically significant effect was observed for pain frequency at either the end of treatment or the end of follow-up. Although quantitative synthesis was restricted to studies reporting comparable outcome definitions and reporting time units, the pooled estimates remained consistently non-significant, and subgroup analyses did not identify differences according to supplement formulation or probiotic dose. These findings, together with the very low certainty of the evidence, do not support a consistent effect of *L. reuteri* DSM 17938 on pain frequency.

Interpretation of the safety profile was limited because adverse events were insufficiently operationalized across the included trials. Although several studies mentioned adverse events, most did not define what constituted an adverse event, describe the methods used for adverse event surveillance or ascertainment, or specify how severity and causality were assessed. Furthermore, two trials reported the absence of adverse events only among participants receiving *L. reuteri* DSM 17938, without explicitly reporting the corresponding findings for the placebo group [12,28]. Consequently, the absence of reported adverse events should not be interpreted as evidence of an established safety profile, but rather as reflecting important limitations in adverse event definition, ascertainment, and reporting. Similar shortcomings have been documented across randomized controlled trials in other clinical fields [31]. Interestingly, a phase I trial evaluating *L. reuteri* DSM 17938 in a pediatric population without FAPDs prospectively defined adverse events and implemented standardized procedures for active surveillance, severity grading, and clinical adjudication [32], illustrating that more rigorous safety assessment is feasible for this intervention. Accordingly, the certainty of the evidence for adverse events remained low.

Several patient-important outcomes also appeared to be incompletely reported. School absenteeism, additional gastrointestinal symptoms, and rescue medication use were evaluated in multiple trials; however, quantitative results were frequently unavailable, selectively reported, or presented only narratively, limiting quantitative synthesis and reducing confidence in the available evidence. In particular, rescue medication use was prespecified in three trials but remained unreported in all of them [9,10,28], whereas parental or caregiver work absenteeism was evaluated by only a single trial despite representing an important outcome for affected families. These findings highlight the need for future trials to prespecify clinically relevant outcomes, adopt standardized outcome definitions, and ensure complete reporting of both efficacy and safety outcomes.

The present review presented strengths that are worth noting as including adherence to Cochrane methodology, outcome-specific risk-of-bias assessments, explicit evaluation of missing results in the synthesis, application of the GRADE approach, and separate analyses according to outcome assessment time points.

Nevertheless, several limitations should be considered when interpreting the findings. First, the available evidence was limited to seven randomized controlled trials, most of which enrolled relatively small numbers of participants, reducing the precision of the available estimates. Second, for pain severity, the secondary end-of-treatment analysis incorporated one additional trial [29], but the pooled estimate proved sensitive to inconsistently reported summary statistics within that study, reducing confidence in the observed short-term effect. Third, for pain frequency, two of the five eligible trials could not be quantitatively synthesized because of incompatible outcome reporting [9,29], resulting in pooled estimates based on only three studies and consequently reducing the amount of information contributing to the meta-analysis. Fourth, evaluation of the safety profile was limited by the insufficient operationalization and reporting of adverse events in the primary studies. Consequently, the available evidence provides only limited information regarding the safety profile of *L. reuteri* DSM 17938 in children with FAPDs, irrespective of the certainty assigned to this outcome. Fifth, although trials using either Rome III or Rome IV diagnostic criteria were eligible, all included trials used Rome III criteria. Therefore, the applicability of the findings to children diagnosed according to Rome IV criteria remains uncertain.

Finally, the evidence for additional outcomes was even more limited than for the primary outcomes. Except for rescue medication use, quantitative synthesis was not possible because of heterogeneous outcome definitions, inconsistent reporting, or missing quantitative results. For rescue medication use, quantitative synthesis was feasible only after imputing the standard deviation for one trial using prespecified assumptions. Although the pooled estimates remained consistent across multiple sensitivity scenarios, the meta-analysis relied on imputed rather than observed variance and should therefore be interpreted with appropriate caution. Moreover, several clinically relevant outcomes were affected by missing results, substantially limiting the information available for clinical decision-making. Although these limitations were reflected in the certainty assessments, they also represent genuine evidence gaps that cannot be resolved through methodological appraisal alone.

## 5. Conclusions

The current evidence does not support a consistent beneficial effect of *L. reuteri* DSM 17938 on pain severity or pain frequency in children with functional abdominal pain disorders. Evidence regarding safety and other patient-important outcomes remains limited because of incomplete and heterogeneous outcome reporting. Overall, the certainty of the evidence across all assessed outcomes ranged from low to very low, limiting confidence in the estimated effects. Future adequately powered, methodologically rigorous randomized controlled trials using standardized outcome definitions and complete reporting are required to determine whether *L. reuteri* DSM 17938 has any measurable effect on these outcomes.

## Figures and Tables

**Table 1:** Characteristics of included studies.

Author, Year	Country	Age	Clinical Population Characteristic	Intervention/Control Sample Size	Intervention Characteristics	Comparison	Duration	Setting	Funding
Romano et al., 2014 [9]	Italy	Population: Children. Age: 6–16 years *Intervention* Mean (sd): 10.2 (2.5) *Control* Mean (sd): 9.6 (0.4)	Functional abdominal pain (FAP) according to Rome III criteria	n = 56 *Intervention:* 30 *Control*: 26	Active compound: *L. reuteri* DSM 17938 10^8^ CFU Presentation: liquid presentation (drops) Administration: oral Dose: 5 drops twice daily	Placebo	Intervention: 4-week Follow-up: 4-week	Pediatric Departments of the Universities of Messina, Palermo, Catania, and the Pediatric Unit of Vittoria.	Probiotic and placebo were donated by BioGaia, Sweden.
Eftekhari et al., 2015 [28]	Iran	Population: Children. Age: 4–16 years *Intervention* Mean (sd): 6.26 (2.10) *Control* Mean (sd): 6.26 (2.61)	Functional abdominal pain (FAP) according to Rome III criteria	n = 80 *Intervention:* 40 *Control*: 40	Active compound: *L. reuteri* DSM 17938 10^8^ CFU (BioGaia^®^) Presentation: liquid presentation (drops) Administration: oral Dose: 5 drops daily	Placebo	Intervention: 4-week Follow-up: 4-week	Gastroenterology clinic of Ayatollah Mousavi hospital	Financial support was provided by vice chancellor for research of Zanjan University of Medical Sciences.
Weizman et al., 2016 [10]	Israel	Population: Children. Age: 6–15 years *Intervention* Mean (sd): 12.2 (2.8) *Control* Mean (sd): 11.7 (3.2)	Abdominal pain-related functional gastrointestinal disorders according to Rome III criteria.	n = 101 *Intervention*: 52 *Control:* 49	Active compound: *L. reuteri* DSM 17938 10^8^ CFU Presentation: Chewable tablet Administration: oral Dose: Once daily	Placebo	Intervention: 4-week Follow-up: 4-week	Pediatric clinics at Soroka Medical Center and at 3 community childcare centers in the Beer-Sheva area.	Study products were donated by BioGaia AB.
Maragkoudaki et al., 2017 [12]	Greece, Slovenia, and Poland	Population: Children. Age: 5–16 years *Intervention* Mean (sd): 9.2 (4.3) *Control* Mean (sd): 9.0 (3.2)	Functional abdominal pain (FAP) according to Rome III criteria	n = 50 *Intervention*: 26 *Control:* 24	Active compound: *L. reuteri* DSM 17938 10^8^ CFU Presentation: chewable Tablet Administration: oral Dose: Two chewable tablet daily	Placebo	Intervention: 4-week Follow-up: 4-week	University hospitals of Athens, Ljubljana, and Warsaw.	Funded by unrestricted grant from BioGaia, Sweden.
Jadrešin et al., 2017 [11]	Croatia	Population: Children. Age: 4–18 years *Intervention* Mean (sd): 10.5 (1.7) *Control* Mean (sd): 7.7 (2.1)	Functional abdominal pain or irritable bowel syndrome according to Rome III criteria.	n = 55 *Intervention:* 26 *Control:* 29	Active compound: *L. reuteri* DSM 17938 (BioGaia^®^) 10^8^ CFU Presentation: 450 mg chewable tablet Administration: oral Dose: Once daily	Placebo	Intervention: 12-week Follow-up: 4-week	Referral Centre for Paediatric Gastroenterology, Children’s Hospital on Zagreb.	Study products were donated by BioGaia.
Rahmani et al., 2020 [29]	Iran	Population: Children. Age: 6–16 years *Intervention* Mean (sd): 7.3 (1.7) *Control* Mean (sd): 7.7 (2.1)	Functional abdominal pain, irritable bowel syndrome, functional dyspepsia or abdominal migraine according to Rome III criteria.	n = 125 *Intervention*: 65 *Control:* 60	Active compound: *L. reuteri* DSM 17938 10^8^ CFU Presentation: chewable tablets Administration: oral Dose: 2 chewable tablets daily	Placebo	Intervention: 4-week Follow-up: Not specified	Gastroenterology clinic	Cooperation and support of Tehran University of Medical Sciences, Tehran, Iran.
Jadrešin et al., 2020 [13]	Croatia	Population: Children. Age: 4–18 years. *Intervention* Median (range): 10.1 (5.4–17.1) *Control* Median (range): 10.6 (5–17)	Functional abdominal pain or irritable bowel syndrome according to Rome III criteria.	n = 46 *Intervention*: 24 *Control:* 22	Active compound: *L. reuteri* DSM 17938 (BioGaia^®^) 10^8^ CFU Presentation: 450 mg chewable tablet Administration: oral Dose: Once daily	Placebo	Intervention: 12-week Follow-up: 4-week	Referral Centre for Paediatric Gastroenterology, Children’s Hospital on Zagreb.	Study products were donated by BioGaia.

**Table 2:** Summary of evidence synthesis strategies for the primary outcomes.

Outcome	Number of RCT Assessing the Outcome	Evidence Synthesis	Additional Analyses
Pain severity	7	Meta-analysis ^§^: End of follow-up: 6 RCT End of treatment: 7 RCT	Sensitivity analysis * Subgroup analyses ^†^
Pain frequency	5	Meta-analysis ^§^: End of follow-up: 3 RCT End of treatment: 3 RCT	Subgroup analyses ^‡^
Quality of life	0	Not applicable	Not applicable
Adverse events	6	Narrative synthesis	Not applicable

^§^ The end-of-follow-up analyses were considered the primary analyses for pain severity and pain frequency. * Sensitivity analysis for the end-of-treatment period using the alternative values reported in the text rather than the corresponding table for one trial (Rahmani 2020 [29]). ^†^ Subgroup analyses according to follow-up duration, supplement presentation and probiotic dose. ^‡^ Subgroup analyses according to supplement presentation and probiotic dose.

**Table 3:** Availability of outcome results across included studies for assessment of risk of bias due to missing results in the synthesis.

Study	Sample Size (*L. reuteri*)	Sample Size (Placebo)	Pain Severity	Pain Frequency	Adverse Events	School Absenteeism	Work Absenteeism	Additional GI Symptoms Frequency	Additional GI Symptoms Severity	Use of Drugs
Romano et al., 2014 ^a^ [9]	30	26	✓	✓	✓	—	—	X	X	X
Eftekhari et al., 2015 ^b^ [28]	40	40	✓	✓	✓	—	—	X	X	X
Weizman et al., 2016 ^c^ [10]	52	49	✓	✓	✓	✓	—	✓	X	X
Maragkoudaki et al., 2017 [12]	26	24	✓	✓	✓	✓	✓	✓	✓	✓
Jadrešin et al., 2017 ^d^ [11]	26	29	✓	—	✓	X	—	—	—	—
Jadrešin et al., 2020 ^e^ [13]	24	22	✓	—	✓	X	—	—	—	—
Rahmani et al., 2020 ^f^ [29]	65	60	✓	✓	—	X	—	—	—	✓

✓, Study results available for inclusion; —, No study result is available for inclusion, (probably) because the outcome was not assessed in the study; X, No study result is available for inclusion, (probably) because the *p* value, magnitude or direction of the results generated were considered unfavorable by the study investigators; ^a^ Romano 2014 [9] included in the methods section other gastrointestinal symptoms (named in the publication as “any other symptoms”) and the use of drugs. No report was provided for use of drugs. The trial reported no “other unexpected symptoms” in the study. ^b^ Eftekhari 2015 [28] included in the methods section other gastrointestinal symptoms (named in the publication as “other symptoms”) and drugs side effects. No report was provided for use of drugs. The trial reported in the abstract section that “there were no significant differences in associated symptoms”. ^c^ Weizman 2016 [10] reported in the abstract that “there was no difference in other gastrointestinal symptoms except for a lower incidence of perceived abdominal distention and bloating, favoring *L reuteri*.” The trial reported drug use for abdominal pain only in the baseline characteristics table. ^d^ Jadrešin 2017 [11] included school absenteeism as an exploratory outcome and only reported that the groups did not differ. ^e^ Jadrešin 2020 [13] included school absenteeism as an exploratory outcome and only reported that the groups did not differ. ^f^ Rahmani 2020 [29] considered as an outcome the number of days affected by day-to-day activities and stated that these activities included school absenteeism. The trial reported narratively that “the number of days of disturbance in daily activities, such as the absence of children from the school, was also reduced in the treatment group” without providing any numeric data.

**Table 4:** GRADE certainty of evidence of main outcomes.

*Lactobacillus Reuteri* Compared to Placebo for Pediatric Functional Abdominal Pain Disorder					
Patient or population: Pediatric functional abdominal pain disorder Intervention: *Lactobacillus Reuteri* Comparison: Placebo					
Outcomes	Anticipated Absolute Effects * (95% CI)		№ of Participants (Studies)	Certainty of the Evidence (GRADE)	Comments
	Risk with Placebo	Risk with *Lactobacillus Reuteri*			
Pain severity at the end-of-follow-up assessed with: Pain scales (Wong-Baker and Hicks’s Faces Pain Scale Revised) follow-up: range 8 weeks to 16 weeks	-	SMD **0.58 SD lower** (1.32 lower to 0.16 higher)	386 (6 RCTs)	⨁◯◯◯ Very low ^a–c^	The evidence is very uncertain about the effect of *Lactobacillus Reuteri* on pain severity at end of follow-up.
Pain severity at the end-of-treatment assessed with: Pain scales (Wong-Baker and Hicks’s Faces Pain Scale Revised) follow-up: range 4 weeks to 16 weeks	-	SMD **0.49 SD lower** (0.93 lower to 0.06 lower)	513 (7 RCTs)	⨁◯◯◯ Very low ^d–f^	The evidence is very uncertain about the effect of *Lactobacillus Reuteri* on pain severity at end of treatment.
Pain frequency at the end-of-follow-up assessed with: Number of episodes per week follow-up: 8 weeks	The mean pain frequency at the end-of-follow-up was **0** episodes per week ^g^	MD **0.15 episodes per week lower** (1.18 lower to 0.87 higher)	229 (3 RCTs) ^g–i^	⨁◯◯◯ Very low ^j–l^	*Lactobacillus Reuteri* may reduce/have little to no effect on pain frequency at the end of follow-up but the evidence is very uncertain.
Pain frequency at the end-of-treatment assessed with: Number of episodes per week follow-up: 4 weeks	The mean pain frequency at the end-of-treatment was **0** episodes per week ^m^	MD **0.6 episodes per week lower** (2.01 lower to 0.81 higher)	231 (3 RCTs) ^i,m,n^	⨁◯◯◯ Very low ^k,l,o^	*Lactobacillus Reuteri* may reduce/have little to no effect on pain frequency at the end of treatment but the evidence is very uncertain.
Adverse events follow-up: range 8 weeks to 16 weeks			(6 RCTs)	⨁⨁◯◯ Low ^p^	The evidence is very uncertain about the effect of *Lactobacillus Reuteri* on adverse events.
* **The risk in the intervention group** (and its 95% confidence interval) is based on the assumed risk in the comparison group and the **relative effect** of the intervention (and its 95% CI). **CI:** confidence interval; **MD:** mean difference; **SMD:** standardised mean difference					
**GRADE Working Group grades of evidence** **High certainty:** We are very confident that the true effect lies close to that of the estimate of the effect. **Moderate certainty:** We are moderately confident in the effect estimate: the true effect is likely to be close to the estimate of the effect, but there is a possibility that it is substantially different. **Low certainty:** Our confidence in the effect estimate is limited: the true effect may be substantially different from the estimate of the effect. **Very low certainty:** We have very little confidence in the effect estimate: the true effect is likely to be substantially different from the estimate of effect.					

Explanations. ^a.^ Five out of six studies were judged as low risk of bias; ^b.^ Considerable heterogeneity (I^2^ = 89.3%) was present and not explained by sub group analysis; ^c.^ The confidence interval crossed one threshold (defined as 0.2 SD). The optimal information size (OIS) predefined as 400, was not met; ^d.^ Five out of seven studies were judged as low risk of bias; ^e.^ Considerable heterogeneity (I^2^ = 82%) was present and not explained by sub group analysis; ^f.^ The confidence interval crossed one threshold (defined as 0.2 SD). The optimal information size (OIS) predefined as 400, was met; ^g.^ The total number of participants in the 5 studies assessing the outcome was 198; ^h.^ The total number of participants in the 5 studies assessing the outcome was 212; ^i.^ A total of 5 studies assessed the outcome. Only 3 studies were included in the meta-analysis because of unit discrepancy limited comparability; ^j.^ Moderate heterogeneity (I^2^ = 60.4%) was present; ^k.^ The confidence interval crossed one threshold (defined as 1.08 [0.2 SD of SD = 5.42]). The optimal information size (OIS) predefined as 400, was not met; ^l.^ Two studies (Romano 2014 [9] and Rahmani 2020 [29]) were not included in the meta-analysis as they reported number of episodes in different time unit. Romano 2014 [9] reported episodes per day, whereas Rahmani 2020 [29] did not specify the time in which episodes were assessed. This avoided their incorporation in the quantitative synthesis; ^m.^ The total number of participants in the 5 studies assessing the outcome was 199; ^n.^ The total number of participants in the 5 studies assessing the outcome was 213; ^o.^ Considerable heterogeneity (I^2^ = 95.2%) was present and not explained by sub group analysis; ^p.^ Five out of six studies showed high risk of bias. Outcome definition was not provided. Results were provided only narratively as no occurrence.

**Table 5:** GRADE certainty of evidence of additional outcomes.

*Lactobacillus Reuteri* Compared to Placebo for Pediatric Functional Abdominal Pain Disorder					
Patient or population: pediatric functional abdominal pain disorder Intervention: *Lactobacillus Reuteri* Comparison: Placebo					
Outcomes	Anticipated Absolute Effects * (95% CI)		№ of Participants (Studies)	Certainty of the Evidence (GRADE)	Comments
	Risk with Placebo	Risk with *Lactobacillus Reuteri*			
School absenteeism follow-up: range 4 weeks to 16 weeks	The mean school absenteeism was **0**	not pooled	367 (5 RCTs) ^a^	⨁◯◯◯ Very low ^b–d^	The evidence is very uncertain about the effect of *Lactobacillus Reuteri* on school absenteeism.
Work absenteeism at the end-of-follow-up assessed with: Number of days lost of work per week follow-up: 8 weeks	The mean work absenteeism at the end-of-follow-up was **0** days per week	MD **0.12 days per week lower** (0.35 lower to 0.11 higher)	48 (1 RCT)	⨁⨁◯◯ Low ^c^	*Lactobacillus Reuteri* may result in little to no difference in work absenteeism.
Additional gastrointestinal symptoms frequency follow-up: 8 weeks	The mean additional gastrointestinal symptoms frequency was **0**	not pooled	277 (4 RCTs) ^e^	⨁◯◯◯ Very low ^c,f–h^	The evidence is very uncertain about the effect of *Lactobacillus Reuteri* on additional gastrointestinal symptoms frequency.
Additional gastrointestinal symptoms severity follow-up: 8 weeks	The mean additional gastrointestinal symptoms severity was **0**	not pooled	277 (4 RCTs) ^i^	⨁◯◯◯ Very low ^c,j,k^	The evidence is very uncertain about the effect of *Lactobacillus Reuteri* on additional gastrointestinal symptoms severity.
Healthcare utilization (as use of drugs) at the end-of-treatment assessed with: days per week follow-up: range 4 weeks to 8 weeks	The mean healthcare utilization (as use of drugs) at the end-of-treatment was **0** days per week ^l^	MD **0.03 days per week higher** (0.01 lower to 0.08 higher)	175 (2 RCTs) ^l–n^	⨁◯◯◯ Very low ^c,o,p^	*Lactobacillus Reuteri* may increase/have little to no effect on use of drugs but the evidence is very uncertain.
* **The risk in the intervention group** (and its 95% confidence interval) is based on the assumed risk in the comparison group and the **relative effect** of the intervention (and its 95% CI). **CI:** confidence interval; **MD:** mean difference; **SMD:** standardised mean difference					
**GRADE Working Group grades of evidence** **High certainty:** We are very confident that the true effect lies close to that of the estimate of the effect. **Moderate certainty:** We are moderately confident in the effect estimate: the true effect is likely to be close to the estimate of the effect, but there is a possibility that it is substantially different. **Low certainty:** Our confidence in the effect estimate is limited: the true effect may be substantially different from the estimate of the effect. **Very low certainty:** We have very little confidence in the effect estimate: the true effect is likely to be substantially different from the estimate of effect.					

Explanations: ^a^ Out of five studies, only two provided quantitative results. Three studies reported results as no difference between groups or in favor of treatment without quantitative information; ^b^ Of the two studies providing quantitative results, one showed some concerns in the bias selection of result domain; ^c^ The optimal information size (OIS) predefined as 400, was not met; ^d^ Three out of five studies assessing the outcome provided only narrative results indicating no differences between groups or reduction in absenteeism in favor of treatment (without any quantitative results); ^e^ Out of four studies only two provided quantitative results. Two studies reported results as no difference between groups; ^f^ Out of the four studies, two provided quantitative results (Weizman 2016 [10] and Maragkoudaki 2017 [12]). Of these studies, one had high risk of bias and the other showed some concerns; ^g^ For the two studies providing quantitative results, the effect showed a reduction frequency in the *L. reuteri* group. Weizman 2016 [10]: Estimated mean difference = −1.5 (95% CI −2.10 to −0.90) episodes/week. Maragkoudaki 2017 [12]: Estimated risk difference = −0.29 (95% CI −0.54 to −0.04) of nausea frequency; ^h^ Two studies, Romano 2014 [9] and Eftekhari 2015 [28], included the outcome in their methods section. No quantitative data was provided and results were only presented as “no difference” or “no other unexpected symptoms”; ^i^ Out of four studies only one provided quantitative results. Two studies considered the outcome in their methods section and reported results as no difference between groups. One study reported results of the outcome only in the abstract that as no difference in other gastrointestinal symptoms; ^j^ Out of the four studies, only one provided quantitative results (Maragkoudaki 2017 [12]). This study had high risk of bias; ^k^ Out of the four studies, only one provided quantitative results (Maragkoudaki 2017 [12]). Of these studies, one had high risk of bias and the other showed some concerns. The other 3 studies only reported results narratively no differences between groups; ^l^ The total number of participants in the 5 studies assessing the outcome was 196; ^m^ The total number of participants in the 5 studies assessing the outcome was 208; ^n^ A total of 5 studies assessed the outcome. Only 2 studies provided quantitative results and were meta analyzed, while 3 studies did not report any result (quantitative or qualitative) of the outcome; ^o^ Of the meta-analyzed studies, one had high risk of bias while the other showed some concerns; ^p^ A total of 5 studies assessed the outcome, but 3 of them did not report any result.

## Data Availability

The study protocol was registered at Universidad Científica del Sur (Protocol No. POS-119-2024-01072) and in PROSPERO (CRD42024618099). The complete search strategies and additional methodological information are provided in the Supplementary Materials. Data extraction files, analysis code, and other materials generated during this systematic review are available from the corresponding author upon reasonable request.

## Supplementary Materials

The following supporting information can be downloaded at https://www.mdpi.com/article/10.3390/nu18172795/s1. Supplementary Materials S1: Search strategies; Supplementary Materials S2: Full-text studies excluded after eligibility assessment and reasons for exclusion; Supplementary Materials S3: Risk of bias assessment: domain-level judgements and explanations; Supplementary Materials S4: Supplementary methods: data extraction and derivation of quantitative estimates; Figure S1: Pain severity at the end-of-treatment sensitivity analysis forest plot; Figure S2: Pain severity at the end-of-follow-up trial duration subgroup forest plot; Figure S3: Pain severity at the end-of-follow-up *Lactobacillus reuteri* DSM 17938 dose per day subgroup forest plot; Figure S4: Pain severity at the end-of-follow-up *Lactobacillus reuteri* DSM 17938 supplement presentation subgroup forest plot; Figure S5: Pain frequency at the end-of-follow-up *Lactobacillus reuteri* DSM 17938 dose per day subgroup forest plot; Figure S6: Pain frequency at the end-of-follow-up *Lactobacillus reuteri* DSM 17938 supplement presentation subgroup forest plot; Figure S7: Use of drugs at the end-of-treatment primary meta-analysis with near-zero variance imputation; Figure S8: Use of drugs at the end-of-treatment sensitivity meta-analysis with borrowed variance imputation; Figure S9: Use of drugs at the end-of-treatment sensitivity meta-analysis with half of the borrowed variance imputation; Supplementary Table S1: Quantitative synthesis of rescue medication use (use of drugs) according to analytical scenario; Supplementary Table S2: GRADE evidence profile of all outcomes; Supplementary Table S3: PRISMA 2020 Checklist.

## Abbreviations

The following abbreviations are used in this manuscript:

CENTRAL	Cochrane Central Register of Controlled Trials
CI	Confidence Interval
GRADE	Grading of Recommendations Assessment, Development and Evaluation
I^2^	Inconsistency statistic
MD	Mean Difference
MeSH	Medical Subject Headings
OIS	Optimal Information Size
PRISMA	Preferred Reporting Items for Systematic Reviews and Meta-Analyses
PROSPERO	International Prospective Register of Systematic Reviews
RCT	Randomized Controlled Trial
REML	Restricted Maximum Likelihood
RoB	Risk of Bias
SD	Standard Deviation
SE	Standard Error
