# Supplementary material for: Efficacy and Safety of Lactobacillus reuteri DSM 17938 in Pediatric Functional Abdominal Pain Disorders: A Systematic Review and Meta-Analysis of Randomized Controlled Trials
Source: Nutrients. 2026 Aug 26;18(17):2795. doi: 10.3390/nu18172795 (PMC13567511; doi:10.3390/nu18172795)
Supplement: Supplementary file 1 [file nutrients-18-02795-s001.zip › nutrients-4445915-supplementary.pdf]

Supplementary Materials

Efficacy and Safety of *Lactobacillus reuteri* DSM 17938 in Pediatric Functional Abdominal Pain Disorders: A Systematic Review and Meta-analysis of Randomized Controlled Trials

Table of Contents

Supplementary materials S1. Search strategies……………………………………..….2

Supplementary materials S2. Full-text studies excluded after eligibility assessment and reasons for exclusion …...………………………...……………………………6

Supplementary materials S3. Risk of Bias assessment: Domain-level judgements and explanations…………………………………………………………………..…7

Supplementary materials S4. Supplementary Methods: Data extraction and derivation of quantitative estimates…………………………………………………20

Supplementary Figure S1…………………………………………………………24

Supplementary Figure S2…………………………………………………………24

Supplementary Figure S3…………………………………………………………25

Supplementary Figure S4…………………………………………………………25

Supplementary Figure S5…………………………………………………………26

Supplementary Figure S6…………………………………………………………26

Supplementary Figure S7…………………………………………………………27

Supplementary Figure S8…………………………………………………………27

Supplementary Figure S9…………………………………………………………27

Supplementary Table S1……………………………………………………………28

Supplementary Table S2……………………………………………………………29

Supplementary Table S3……………………………………………………………32

Supplementary materials S1. Search strategies.

Database: PubMed

Date of the search: April 8, 2026

Search	Terms	Results
#1	Abdominal Pain[Mesh]	35,403
#2	Abdom* Pain*[tiab]	84,804
#3	Colic*[tiab]	17,436
#4	Acute Abdom*[tiab]	16,191
#5	Abdom* Distress*[tiab]	157
#6	Ache*[tiab]	30,423
#7	#1 OR #2 OR #3 OR #4 OR #5 OR #6	153,085
#8	Limosilactobacillus Reuteri[Mesh]	1,399
#9	Lactobacillus Reuteri[tiab]	2,384
#10	Limosilactobacillus Reuteri[tiab]	681
#11	DSM 17938*[tiab]	300
#12	DSM17938*[tiab]	33
#13	#8 OR #9 OR #10 OR #11 OR #12	3,115
#14	#7 AND #13	144

Database: Cochrane Library

Date of the search: April 8, 2026

Search	Terms	Results
#1	MH Abdominal Pain	855
#2	(Abdom* NEAR/1 Pain*):ti,ab,kw	16,639

Search	Terms	Results
#3	Colic*:ti,ab,kw	2,416
#4	(Acute NEAR/1 Abdom*):ti,ab,kw	427
#5	(Abdom* NEAR/1 Distress*):ti,ab,kw	29
#6	Ache*:ti,ab,kw	1,957
#7	#1 OR #2 OR #3 OR #4 OR #5 OR #6	21,641
#8	MH Limosilactobacillus Reuteri	8
#9	(Lactobacillus NEAR/1 Reuteri):ti,ab,kw	776
#10	(Limosilactobacillus NEAR/1 Reuteri):ti,ab,kw	298
#11	DSM-17938*:ti,ab,kw	241
#12	DSM7938*:ti,ab,kw	0
#13	#8 OR #9 OR #10 OR #11 OR #12	874
#14	#7 AND #13	151

Database: Embase (Ovid)

Date of the search: April 8, 2026

Search	Terms	Results
1	exp abdominal pain/	302,480
2	(Abdom* adj1 Pain*).ti,ab.	156,966
3	Colic*.ti,ab.	22,878
4	(Acute adj1 Abdomen).ti,ab.	10,411
5	(Abdom* adj1 Distress*).ti,ab.	311
6	Ache*.ti,ab.	38,941

Search	Terms	Results
7	1 or 2 or 3 or 4 or 5 or 6	396,872
8	exp Lactobacillus reuteri/	5,195
9	(Lactobacillus adj1 Reuteri).ti,ab.	2,929
10	(Limosilactobacillus adj1 Reuteri).ti,ab.	668
11	DSM-17938*.ti,ab.	442
12	DSM17938*.ti,ab.	57
13	8 or 9 or 10 or 11 or 12	5,717
14	7 and 13	366

Database: Web of Science Core Collection

Date of the search: April 8, 2026

Search	Terms	Results
#1	(TS=Abdominal Pain OR TI=(Abdom* NEAR/1 Pain*) OR AB=(Abdom* NEAR/1 Pain*) OR TI=Colic* OR AB=Colic* OR TI=(Acute NEAR/1 Abdom*) OR AB=(Acute NEAR/1 Abdom*) OR TI=(Abdom* NEAR/1 Distress*) OR AB=(Abdom* NEAR/1 Distress*) OR TI=Ache* OR AB=Ache*) AND (TS=Limosilactobacillus Reuteri OR TI=(Lactobacillus NEAR/1 Reuteri) OR AB=(Lactobacillus NEAR/1 Reuteri) OR TI=(Limosilactobacillus NEAR/1 Reuteri) OR AB=(Limosilactobacillus NEAR/1 Reuteri) OR TI=DSM-17938* OR AB=DSM-17938* OR TI=DSM17938* OR AB=DSM17938*)	123

Database: Scopus

Date of the search: April 8, 2026

Search	Terms	Results
#1	(INDEXTERMS("Abdominal Pain") OR TITLE-ABS("Abdominal Pain*") OR TITLE-ABS("Abdomen Pain*") OR TITLE-	165

Search	Terms	Results
	ABS(colic*) OR TITLE-ABS("Acute Abdom*") OR TITLE-ABS("Abdom* Distress*") OR TITLE-ABS(ache*)) AND (INDEXTERMS("Limosilactobacillus Reuteri") OR TITLE-ABS("Lactobacillus Reuteri") OR TITLE-ABS("Limosilactobacillus Reuteri") OR TITLE-ABS("DSM 17938*") OR TITLE-ABS(dsm17938*))	

Database: CINAHL (EBSCO)

Date of the search: April 8, 2026

Search	Terms	Results
#1	(MH "Abdominal Pain+" OR XB (Abdom* N1 Pain*) OR XB Colic* OR XB (Acute N1 Abdom*) OR XB (Abdom* N1 Distress*) OR XB Ache*) AND (XB (Lactobacillus N1 Reuteri) OR XB (Limosilactobacillus N1 Reuteri) OR XB DSM-17938* OR XB DSM17938*)	56

Database: LILACS

Date of the search: April 8, 2026

Search	Terms	Results
#1	((mh:(abdominal pain) OR (abdominal pain*) OR (dolor abdominal) OR (dor abdominal) OR (colic*) OR (acute abdom*) OR (abdominal agudo) OR (abdome agudo) OR (abdominal distress*) OR (abdomen distress) OR (malestar abdom*) OR (desconforto abdom*) OR (ache*) OR (calambres abdominales) OR (calambres abdominais)) AND (mh:(limosilactobacillus reuteri) OR (lactobacillus reuteri) OR (limosilactobacillus reuteri) OR (dsm 17938*) OR (dsm17938*))) AND db:("LILACS" OR "BINACIS" OR "BRISA") AND instance:"lilacsplus"	4

Supplementary materials S2. Full-text studies excluded after eligibility assessment and reasons for exclusion.

Study	Reason for exclusion	Link or DOI
Jadresin, 2016	Wrong publication type	10.1097/01.mpg.0000484500.48517.e7
LRCT2014083018971N, 2014	Wrong publication type	https://www.cochranelibrary.com/central/doi/10.1002/central/CN-01860189/full
Maragkoudaki, 2016	Wrong publication type	10.1097/01.mpg.0000484500.48517.e7
Motamed, 2019	Wrong publication type	10.1097/MPG.0000000000002403
NCT, 2012	Wrong publication type	https://www.cochranelibrary.com/central/doi/10.1002/central/CN-01538486/full
NCT, 2012	Wrong publication type	https://www.cochranelibrary.com/central/doi/10.1002/central/CN-01503142/full.

Supplementary materials S3. Risk of Bias assessment: Domain-level judgements and explanations.

Outcome: Pain severity

**Domain 1: Randomization process**		
**Study**	**Judgement**	**Explanation**
Romano 2014	Low	The randomization process was adequately described and no baseline imbalances suggested concerns regarding randomization.
Eftekhari 2015	Low	The randomization process was probably adequate, and baseline characteristics did not suggest concerns regarding randomization.
Weizman 2016	Low	The randomization process was adequately described, although baseline characteristics were reported only for protocol completers.
Maragkoudaki 2017	Low	The randomization process was adequately described, and no concerns regarding baseline imbalance were identified.
Jadresin 2017	Low	Randomization was generated using dedicated randomization software, and allocation concealment was achieved using sequentially numbered, opaque, sealed envelopes prepared by an independent person.
Jadresin 2020	Low	Randomization was generated using dedicated randomization software, and allocation concealment was achieved using sequentially numbered, opaque, sealed envelopes prepared by an independent person.

Rahmani 2020	Some concerns	Randomization was adequately described, but allocation concealment was unclear.
**Domain 2: Deviations from intended interventions**		
**Study**	**Judgement**	**Explanation**
Romano 2014	Some concerns	The study was double-blinded, but four randomized participants were excluded from the analysis due to poor compliance, suggesting a per-protocol analysis. Exclusions were balanced across groups.
Eftekhari 2015	Low	Participants and physicians were blinded to treatment allocation, and all randomized participants were included in the analysis.
Weizman 2016	Low	Participants and study personnel were blinded to treatment allocation, and the primary analysis was conducted in an intention-to-treat population comprising all randomized participants.
Maragkoudaki 2017	Low	The double-blind design minimized deviations from intended interventions, and the statistical analysis included all available outcome data during follow-up.
Jadresin 2017	Low	Participants and study personnel were blinded, and all analyses were conducted on an intention-to-treat basis.
Jadresin 2020	Low	Participants and study personnel were blinded, and all analyses were conducted on an intention-to-treat basis.
Rahmani 2020	Some concerns	Participant blinding was likely maintained, but blinding of study personnel was insufficiently described.
**Domain 3: Missing outcome data**		
**Study**	**Judgement**	**Explanation**
Romano 2014	Low	Outcome data were available for nearly all participants, and losses due to poor compliance were small and balanced between groups.
Eftekhari 2015	Low	Although participant flow was not explicitly reported, the number of analyzed participants appeared consistent with the number randomized, suggesting that outcome data were available for nearly all participant.
Weizman 2016	Low	A small and balanced proportion of participants did not complete treatment. An intention-to-treat analysis was reported, suggesting limited risk of bias due to missing outcome data.
Maragkoudaki 2017	Low	Outcome data were available for nearly all randomized participants, with only a small number of participants not contributing endpoint data.
Jadresin 2017	Low	Outcome data were available for nearly all participants, with no important concerns regarding missing data.
Jadresin 2020	Low	Outcome data were available for nearly all participants, with no important concerns regarding missing data.

Rahmani 2020	Low	Outcome data were available for nearly all participants, with no important concerns regarding missing data.
**Domain 4: Measurement of the outcome**		
**Study**	**Judgement**	**Explanation**
Romano 2014	Low	Pain severity was measured using a standardized symptom scale, and blinded outcome assessment minimized the likelihood of differential measurement between groups.
Eftekhari 2015	Low	Outcome measurement appeared comparable between groups, although details regarding the timing of pain assessment were not fully reported.
Weizman 2016	Low	Pain severity was assessed using an appropriate outcome measure. Although details regarding outcome assessors were limited, the study was reported as double-blind, making differential outcome measurement between groups unlikely.
Maragkoudaki 2017	Low	Pain severity was measured using the same procedures in both groups. Although the scale description was not fully detailed, the double-blind design made differential outcome assessment unlikely.
Jadresin 2017	Low	Outcome measurement was likely comparable between groups, with limited risk of differential assessment.
Jadresin 2020	Low	Outcome measurement was likely comparable between groups, with limited risk of differential assessment.
Rahmani 2020	Some concerns	Pain severity is a subjective outcome, and insufficient information was provided regarding blinding of outcome assessors.
**Domain 5: Selection of the reported result**		
**Study**	**Judgement**	**Explanation**
Romano 2014	Some concerns	No trial registration or prespecified analysis plan was available, limiting assessment of whether the reported pain severity result was selected from multiple eligible analyses.
Eftekhari 2015	Low	Pain severity was clearly prespecified and operationalized in the Methods section, with a defined measurement scale and follow-up assessment procedures. Although no trial registration was available, no evidence of selective reporting was identified..
Weizman 2016	Low	Pain severity was prespecified in the study protocol and trial registration. No evidence suggested selective reporting of outcome measurements or analyses.
Maragkoudaki 2017	Low	Pain severity was prespecified in the trial registration, and no evidence suggested selective reporting of outcome measurements or analyses.
Jadresin 2017	Low	Pain severity was prespecified, and no evidence of selective reporting was identified.

Jadresin 2020	Low	Pain severity was prespecified, and no evidence of selective reporting was identified.
Rahmani 2020	Some concerns	Although pain intensity was assessed using the WBFPRS, the timing of assessments and prespecified analytical approach were not clearly described.

Outcome: Pain frequency

**Domain 1: Randomization process**		
**Study**	**Judgement**	**Explanation**
Eftekhari 2015	Low	The randomization process was probably adequate, and baseline characteristics did not suggest concerns regarding randomization.
Maragkoudak 2017	Low	The randomization process was adequately described, and no concerns regarding baseline imbalance were identified.
Weizman 2016	Low	The randomization process was adequately described, although baseline characteristics were reported only for protocol completers.
Romano 2014	Low	The randomization process was adequately described and no baseline imbalances suggested concerns regarding randomization.
Rahmani 2020	Some concerns	Randomization was adequately described, but allocation concealment was unclear.
**Domain 2: Deviations from intended interventions**		
**Study**	**Judgement**	**Explanation**
Eftekhari 2015	Low	Participants and physicians were blinded to treatment allocation, and all randomized participants were included in the analysis.
Maragkoudak 2017	Low	The double-blind design minimized deviations from intended interventions, and the statistical analysis included all available outcome data during follow-up.
Weizman 2016	Low	Participants and study personnel were blinded to treatment allocation, and the primary analysis was conducted in an intention-to-treat population comprising all randomized participants.
Romano 2014	Some concerns	The study was double-blinded, but four randomized participants were excluded from the analysis due to poor compliance, suggesting a per-protocol analysis. Exclusions were balanced across groups.
Rahmani 2020	Some concerns	Participant blinding was likely maintained, but blinding of study personnel was insufficiently described.
**Domain 3: Missing outcome data**		
**Study**	**Judgement**	**Explanation**

Eftekhari 2015	Low	Outcome data for pain frequency appeared available for nearly all participants, based on consistent numbers reported at baseline and follow-up.
Maragkoudak 2017	Low	Missing outcome data were unlikely to meaningfully affect the results, although the analyzed sample for this outcome was not explicitly reported.
Weizman 2016	Low	A small and balanced proportion of participants did not complete treatment. Although the handling of missing data was not fully described, an intention-to-treat analysis was reported.
Romano 2014	Low	Outcome data were available for nearly all participants, and losses due to poor compliance were small and balanced between groups.
Rahmani 2020	Low	Outcome data were available for nearly all participants, with no important concerns regarding missing data.
**Domain 4: Measurement of the outcome**		
**Study**	**Judgement**	**Explanation**
Eftekhari 2015	Low	Pain frequency was reported as episodes per week, but the assessment period was insufficiently described. Blinding of participants and physicians reduced the likelihood of differential outcome measurement.
Maragkoudak 2017	Low	Pain frequency was measured similarly across groups, although the assessment period was not fully specified.
Weizman 2016	Low	Pain frequency was collected through daily self-reporting, although the assessment period used for the reported outcome was not fully described. The blinded design minimized the risk of differential outcome measurement.
Romano 2014	Low	Pain frequency was assessed using daily symptom diaries, but the reporting time horizon was not clearly specified.
Rahmani 2020	Some concerns	Pain frequency was assessed similarly across groups, but limited information regarding outcome assessment and assessor blinding resulted in some concerns about outcome measurement.
**Domain 5: Selection of the reported result**		
**Study**	**Judgement**	**Explanation**
Eftekhari 2015	Some concerns	No trial registration or protocol was available. Pain frequency was insufficiently defined, and neither the intended measurement strategy nor the planned analysis could be verified.
Maragkoudak 2017	Some concerns	The trial registration prespecified pain frequency as an area-under-the-curve outcome, whereas the publication reported episodes per week, preventing verification of consistency between planned and reported analyses.
Weizman 2016	Low	Pain frequency was prespecified in the trial registration as the number of pain episodes assessed over eight weeks, with no important concerns regarding selective reporting.
Romano 2014	Some concerns	No trial registration or protocol was available, and the measurement and analysis of pain frequency were insufficiently described to verify that the reported result was prespecified.

Rahmani 2020	Some concerns	No trial registration or protocol was available, and the reported pain-frequency outcome lacked a clearly defined assessment period, preventing verification of the prespecified measurement and analysis.

Outcome: Adverse events

**Domain 1: Randomization process**		
**Study**	**Judgement**	**Explanation**
Romano 2014	Low	The randomization process was adequately described and no baseline imbalances suggested concerns regarding randomization.
Eftekhari 2015	Low	The randomization process was probably adequate, and baseline characteristics did not suggest concerns regarding randomization.
Weizman 2016	Low	The randomization process was adequately described, although baseline characteristics were reported only for protocol completers.
Maragkoudaki 2017	Low	The randomization process was adequately described, and no concerns regarding baseline imbalance were identified.
Jadresin 2017	Low	Randomization was generated using dedicated randomization software, and allocation concealment was achieved using sequentially numbered, opaque, sealed envelopes prepared by an independent person.
Jadresin 2020	Low	Randomization was generated using dedicated randomization software, and allocation concealment was achieved using sequentially numbered, opaque, sealed envelopes prepared by an independent person.
**Domain 2: Deviations from intended interventions**		
**Study**	**Judgement**	**Explanation**
Romano 2014	Low	Although the algorithm yields "Some concerns", the small number of balanced losses (6.7%), the double-blind design, and the absence of evidence that missingness was related to adverse events make it unlikely that deviations from intended interventions biased the estimated effect.
Eftekhari 2015	Low	Participants and physicians were blinded to treatment allocation, and all randomized participants were included in the analysis.
Weizman 2016	Low	Participants and study personnel were blinded to treatment allocation, and the primary analysis was conducted in an intention-to-treat population comprising all randomized participants.
Maragkoudaki 2017	Low	The double-blind design minimized deviations from intended interventions, and the statistical analysis included all available outcome data during follow-up.
Jadresin 2017	Low	Participants and study personnel were blinded, and all analyses were conducted on an intention-to-treat basis.

Jadresin 2020	Low	Participants and study personnel were blinded, and all analyses were conducted on an intention-to-treat basis.
**Domain 3: Missing outcome data**		
**Study**	**Judgement**	**Explanation**
Romano 2014	Low	Although adverse events were reported only narratively, the proportion of missing outcome data was small and balanced across groups (2 participants lost per arm due to poor compliance). Therefore, risk of bias arising from missing outcome data was judged as low.
Eftekhari 2015	Low	Outcome data were probably available for nearly all randomized participants, as efficacy analyses included all randomized participants and no losses to follow-up were reported.
Weizman 2016	Low	Outcome data were probably available for nearly all randomized participants, and losses to follow-up were unrelated to adverse effects.
Maragkoudaki 2017	Low	Outcome data were probably available for nearly all randomized participants, and no evidence suggested that missing data were related to adverse events.
Jadresin 2017	Low	Outcome data were probably available for nearly all randomized participants, and no evidence suggested that missing data were related to adverse events.
Jadresin 2020	Low	Outcome data were probably available for nearly all randomized participants, and no evidence suggested that missing data were related to adverse events.
**Domain 4: Measurement of the outcome**		
**Study**	**Judgement**	**Explanation**
Romano 2014	High	Adverse events were reported without providing a definition or describing the assessment method (prespecified nut not defined outcome).
Eftekhari 2015	High	Adverse events were reported without providing a definition or describing the assessment method (not prespecified outcome).
Weizman 2016	Low	Adverse events were collected prospectively, but limited information was provided regarding their definition and assessment procedures.
Maragkoudaki 2017	High	Although the trial registry stated that adverse events would be observed and recorded, neither the publication nor the registry described how adverse events were defined or systematically ascertained.
Jadresin 2017	High	Although adverse events were recorded in study diaries and follow-up visits, no definition or classification framework for adverse events was provided.
Jadresin 2020	High	Although adverse events were recorded in study diaries and follow-up visits, no definition or classification framework for adverse events was provided.
**Domain 5: Selection of the reported result**		
**Study**	**Judgement**	**Explanation**

Romano 2014	Some concerns	Adverse events were not prespecified, were reported only through a narrative statement, and there is no information regarding collection procedures, definitions, timing, or analyses. Consequently, the possibility that the reported absence of adverse events reflects selective outcome reporting cannot be excluded and is considered substantial.
Eftekhari 2015	Some concerns	Adverse events were reported only for the intervention group and were not described as a prespecified outcome, raising concerns about selective reporting.
Weizman 2016	Some concerns	Adverse effects were prespecified in the trial registry and reported in the publication, although no detailed prespecified analysis plan was available.
Maragkoudaki 2017	Some concerns	Adverse events were prespecified in the trial registry, but only a narrative safety statement was reported and no detailed prespecified analysis plan was available.
Jadresin 2017	High	Although adverse events were reported in the publication, this outcome was not included among the prespecified outcomes in the trial registry, raising concerns about selective reporting.
Jadresin 2020	High	Although adverse events were reported in the publication, this outcome was not included among the prespecified outcomes in the trial registry, raising concerns about selective reporting.

Outcome: School absenteeism

**Domain 1: Randomization process**		
**Study**	**Judgement**	**Explanation**
Weizman 2016	Low	The randomization process was adequately described, although baseline characteristics were reported only for protocol completers.
Maragkoudaki 2017	Low	The randomization process was adequately described, and no concerns regarding baseline imbalance were identified.
**Domain 2: Deviations from intended interventions**		
**Study**	**Judgement**	**Explanation**
Weizman 2016	Low	Participants and study personnel were blinded to treatment allocation, and the primary analysis was conducted in an intention-to-treat population comprising all randomized participants.
Maragkoudaki 2017	Low	The double-blind design minimized deviations from intended interventions, and the statistical analysis included all available outcome data during follow-up.
**Domain 3: Missing outcome data**		
**Study**	**Judgement**	**Explanation**
Weizman 2016	Low	A small and balanced proportion of participants did not complete treatment. An intention-to-treat analysis was reported, suggesting limited risk of bias due to missing outcome data.
Maragkoudaki 2017	Low	Outcome data were available for nearly all randomized participants, with only a small number of participants not contributing endpoint data.
**Domain 4: Measurement of the outcome**		
**Study**	**Judgement**	**Explanation**
Weizman 2016	Low	The outcome was defined both in the trial register and the publication.
Maragkoudaki 2017	Low	The outcome was defined both in the trial register and the publication as any reduction in days of school absenteeism.
**Domain 5: Selection of the reported result**		
**Study**	**Judgement**	**Explanation**
Weizman 2016	Some concerns	The study did not provide a specific definition and assessment method for school absenteeism measure. The trial register defined the outcome, as "Number of patients with school absenteeism". Potential difference in unit analysis cannot be ruled out.
Maragkoudaki 2017	Low	Trial registration and the methods section outcome definitions were not discordant.

Outcome: Parental or caregiver work absenteeism

**Domain 1: Randomization process**		
**Study**	**Judgement**	**Explanation**
Maragkoudaki 2017	Low	The randomization process was adequately described, and no concerns regarding baseline imbalance were identified.
**Domain 2: Deviations from intended interventions**		
**Study**	**Judgement**	**Explanation**
Maragkoudaki 2017	Low	The double-blind design minimized deviations from intended interventions, and the statistical analysis included all available outcome data during follow-up.
**Domain 3: Missing outcome data**		
**Study**	**Judgement**	**Explanation**
Maragkoudaki 2017	Low	Outcome data were available for nearly all randomized participants, with only a small number of participants not contributing endpoint data.
**Domain 4: Measurement of the outcome**		
**Study**	**Judgement**	**Explanation**
Maragkoudaki 2017	Low	Work absenteeism was measured similarly across groups, although details regarding the assessment period were limited.
**Domain 5: Selection of the reported result**		
**Study**	**Judgement**	**Explanation**
Maragkoudaki 2017	Low	Work absenteeism was clearly prespecified and reported consistently with the trial registration.

Outcome: Additional gastrointestinal symptoms – severity

**Domain 1: Randomization process**		
**Study**	**Judgement**	**Explanation**
Maragkoudaki 2017	Low	The randomization process was adequately described, and no concerns regarding baseline imbalance were identified.
**Domain 2: Deviations from intended interventions**		
**Study**	**Judgement**	**Explanation**
Maragkoudaki 2017	Low	The double-blind design minimized deviations from intended interventions, and the statistical analysis included all available outcome data during follow-up.
**Domain 3: Missing outcome data**		
**Study**	**Judgement**	**Explanation**
Maragkoudaki 2017	Low	Outcome data were available for nearly all randomized participants, with only a small number of participants not contributing endpoint data.
**Domain 4: Measurement of the outcome**		
**Study**	**Judgement**	**Explanation**
Maragkoudaki 2017	Low	Other gastrointestinal symptoms severity was measured similarly across groups, although details regarding the assessment period were limited.
**Domain 5: Selection of the reported result**		
**Study**	**Judgement**	**Explanation**
Maragkoudaki 2017	High	Multiple GSRS assessment time points were prespecified, but detailed results were only presented for the 2-week assessment, raising concerns about selective reporting of outcome measurements.

Outcome: Additional gastrointestinal symptoms – frequency

Study	Judgement	Explanation
**Domain 1: Randomization process**		
**Study**	**Judgement**	**Explanation**
Weizman 2016	Low	The randomization process was adequately described, although baseline characteristics were reported only for protocol completers.
Maragkoudaki 2017	Low	The randomization process was adequately described, and no concerns regarding baseline imbalance were identified.
**Domain 2: Deviations from intended interventions**		
**Study**	**Judgement**	**Explanation**
Weizman 2016	Low	Participants and study personnel were blinded to treatment allocation, and the primary analysis was conducted in an intention-to-treat population comprising all randomized participants.
Maragkoudaki 2017	Low	The double-blind design minimized deviations from intended interventions, and the statistical analysis included all available outcome data during follow-up.
**Domain 3: Missing outcome data**		
**Study**	**Judgement**	**Explanation**
Weizman 2016	Low	A small and balanced proportion of participants did not complete treatment. An intention-to-treat analysis was reported, suggesting limited risk of bias due to missing outcome data.
Maragkoudaki 2017	Low	Outcome data were available for nearly all randomized participants, with only a small number of participants not contributing endpoint data.
**Domain 4: Measurement of the outcome**		
**Study**	**Judgement**	**Explanation**
Weizman 2016	Low	Additional gastrointestinal symptoms were prespecified, but the methods did not clearly describe how these symptoms were assessed or summarized. However, no evidence of differential measurement between groups was present and outcome assessors were blinded..
Maragkoudaki 2017	Low	Other gastrointestinal symptoms frequency was measured similarly across groups, although details regarding the assessment period were limited.
**Domain 5: Selection of the reported result**		
**Study**	**Judgement**	**Explanation**
Weizman 2016	Some concerns	Although other gastrointestinal symptoms were prespecified, the publication did not clearly describe their assessment, timing, or analysis, preventing verification that the reported result corresponded to the prespecified outcome.
Maragkoudaki 2017	High	Multiple GSRS (frequency) assessment time points were prespecified, but detailed results were only presented for the 2-week assessment, raising concerns about selective reporting of outcome measurements.

Outcome: Healthcare utilization – use of drugs

**Domain 1: Randomization process**		
**Study**	**Judgement**	**Explanation**
Maragkoudaki 2017	Low	The randomization process was adequately described, and no concerns regarding baseline imbalance were identified.
Rahmani 2020	Some concerns	Randomization was adequately described, but allocation concealment was unclear.
**Domain 2: Deviations from intended interventions**		
**Study**	**Judgement**	**Explanation**
Maragkoudaki 2017	Low	The double-blind design minimized deviations from intended interventions, and the statistical analysis included all available outcome data during follow-up.
Rahmani 2020	Some concerns	Participant blinding was likely maintained, but blinding of study personnel was insufficiently described.
**Domain 3: Missing outcome data**		
**Study**	**Judgement**	**Explanation**
Maragkoudaki 2017	Low	Outcome data were available for nearly all randomized participants, with only a small number of participants not contributing endpoint data.
Rahmani 2020	Low	Outcome data were available for nearly all participants, with no important concerns regarding missing data.
**Domain 4: Measurement of the outcome**		
**Study**	**Judgement**	**Explanation**
Maragkoudaki 2017	Low	Measurement of drug use was not prespecified or clearly explained in the paper. However, there was no evidence of differential measurements between groups.
Rahmani 2020	Low	Measurement of drug use was prespecified in the paper but not clearly explained in the paper. However, there was no evidence of differential measurements between groups.
**Domain 5: Selection of the reported result**		
**Study**	**Judgement**	**Explanation**
Maragkoudaki 2017	Some concerns	The outcome was not prespecified at trial register. The paper did not explicit how the measurement was conducted or analyzed.
Rahmani 2020	Some concerns	No trial registration. The outcome was prespecified in methods section but did not explicit how the measurement was conducted or analyzed.

Supplementary materials S4. Supplementary Methods: Data extraction and derivation of quantitative estimates

Extraction of graphical data

For the Romano et al. (2014) trial, numerical values for pain severity and pain frequency were not reported in tabular format. Mean values and corresponding standard errors (SEs) were extracted from published figures using PlotDigitizer (https://plotdigitizer.sourceforge.net/). The extracted SEs were converted to standard deviations (SDs) using the following equation:

$$SD = SE \times \sqrt{n}$$

where n represents the number of participants in the corresponding study arm.

Manual estimation of quantitative effect estimates

For outcomes that were not eligible for meta-analysis but provided sufficient summary statistics, quantitative effect estimates were analytically derived from published group-level data. These estimates were used exclusively for narrative synthesis and were not incorporated into quantitative pooling unless explicitly stated.

For continuous outcomes, mean differences (MDs) were calculated as:

$$MD = \bar{X}_I - \bar{X}_C$$

Where $\bar{X}_I$ and $\bar{X}_C$ represent the intervention and control group means, respectively.

The standard error of the mean difference was calculated as:

$$SE = \sqrt{\frac{SD^2_{intervention}}{n_{intervention}} + \frac{SD^2_{control}}{n_{control}}}$$

and the corresponding 95% confidence interval was estimated as:

$$MD \pm t_{0.975,df} \times SE$$

where $t_{0.975,df}$ denotes the two-sided critical value of the t distribution.

For dichotomous outcomes, risk differences (RDs) were calculated as:

$$RD = p_I - p_C$$

where p_I and p_C represent the event proportions in the intervention and control groups, respectively. Exact 95% confidence intervals for the risk difference were calculated using the unconditional exact method.

Derived quantitative estimates were obtained for:

- Pain frequency reported using non-comparable time units or without specification of the assessment period (Romano et al., 2014; Rahmani et al., 2020);
- School absenteeism (Maragkoudaki et al., 2017; Weizman et al., 2016);
- Parental or caregiver work absenteeism (Maragkoudaki et al., 2017);
- Additional gastrointestinal symptom frequency (Weizman et al., 2016; Maragkoudaki et al., 2017);
- Additional gastrointestinal symptom severity (Maragkoudaki et al., 2017).

Additional details on data extraction and quantitative synthesis are presented in Tables A and B below. Table A summarizes the data sources, methods, and derived quantitative estimates for outcomes requiring additional data processing, whereas Table B presents the study-level assessment time points included in each quantitative synthesis.

Table A. Data sources, derivation methods, and quantitative estimates used for evidence synthesis.

Outcome	Study	Time point	Data source	Estimation method	Derived quantitative estimate	Included in MA	Reason for exclusion from MA
Pain frequency	Romano 2014	End of follow-up	Graph (8-week assessment)	PlotDigitizer; SE→SD (SD = SE × √n)	MD = 0.08 (95% CI: −0.36 to 0.52) episodes/day	No	Outcome reported as episodes/day; not comparable with episodes/week
	Rahmani 2020	End of treatment	Table 1	Calculated from published summary statistics	MD = −2.70 (95% CI: −4.16 to −1.24)	No	Time unit for episodes not specified
School absenteeism	Maragkoudaki 2017	End of follow-up	Table 1	Calculated from published summary statistics	MD = −0.11 (95% CI: −0.32 to 0.10) days	No	Single-study evidence
	Weizman 2016	Unclear (treatment or follow-up)	Main text	Calculated from published summary statistics	MD = −0.80 (95% CI: −1.20 to −0.40) days	No	Time point not clearly reported
Parental/caregiver work absenteeism	Maragkoudaki 2017	End of follow-up	Table 1	Calculated from published summary statistics	MD = −0.12 (95% CI: −0.35 to 0.11) days/week	No	Single-study evidence
Additional gastrointestinal symptoms (frequency)	Weizman 2016	Unclear (treatment or follow-up)	Main text	Calculated from published summary statistics	MD = −1.50 (95% CI: −2.10 to −0.90) episodes/week	No	Time point not clearly reported
	Maragkoudaki 2017	Mid-treatment (Week 2)	Table 2	Calculated from published contingency table	RD = −0.29 (95% CI: −0.54 to −0.04)	No	Outcome expressed as nausea frequency (%) at a single time point
Additional gastrointestinal symptoms (severity)	Maragkoudaki 2017	Mid-treatment (Week 2)	Main text	Calculated from published summary statistics	MD = −7.10 (95% CI −13.85 to −0.35) GSRS points	No	Outcome reported only before end of treatment

CI: confidence interval; GSRS: Gastrointestinal Symptom Rating Scale; MD: mean difference; RD: risk difference; SD: standard deviation; SE: standard error; MA: meta-analysis.
Data source refers to the location of the extracted data within the original study report.

Table B. Study-level assessment time points included in quantitative syntheses.

Outcome	Study	End-of-treatment assessment	End-of-follow-up assessment
Pain severity	Eftekhari et al., 2015	Week 4	Week 8
	Jadrešin et al., 2017	Week 12	Week 16
	Jadrešin et al., 2020	Week 12	Week 16
	Maragkoudaki et al., 2017	Week 4	Week 8
	Rahmani et al., 2020	Week 4	—
	Romano et al., 2014	Week 4	Week 8
	Weizman et al., 2016	Week 4	Week 8
Pain frequency	Eftekhari et al., 2015	Week 4	Week 8
	Maragkoudaki et al., 2017	Week 4	Week 8
	Weizman et al., 2016	Week 4	Week 8
Healthcare utilization as use of drugs	Maragkoudaki et al., 2017	Week 4	Week 8
	Rahmani et al., 2020	Week 4	—

Assessment time points are reported as weeks from the start of treatment. —, study did not contribute to the corresponding quantitative synthesis.

Figure S1. Pain severity at the end-of-treatment sensitivity analysis forest plot.

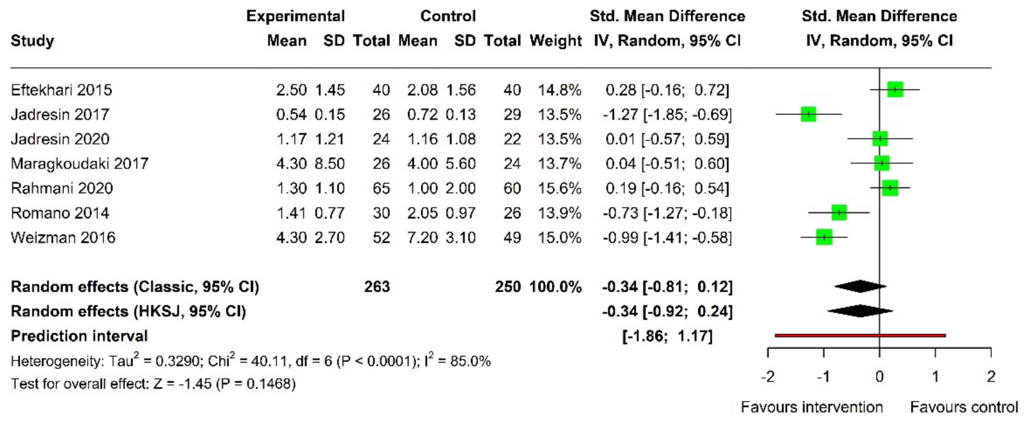

Note: For Rahmani, 2020 meand and SD ere extracted from the text results

Figure S2. Pain severity at the end-of-follow-up trial duration subgroup forest plot.

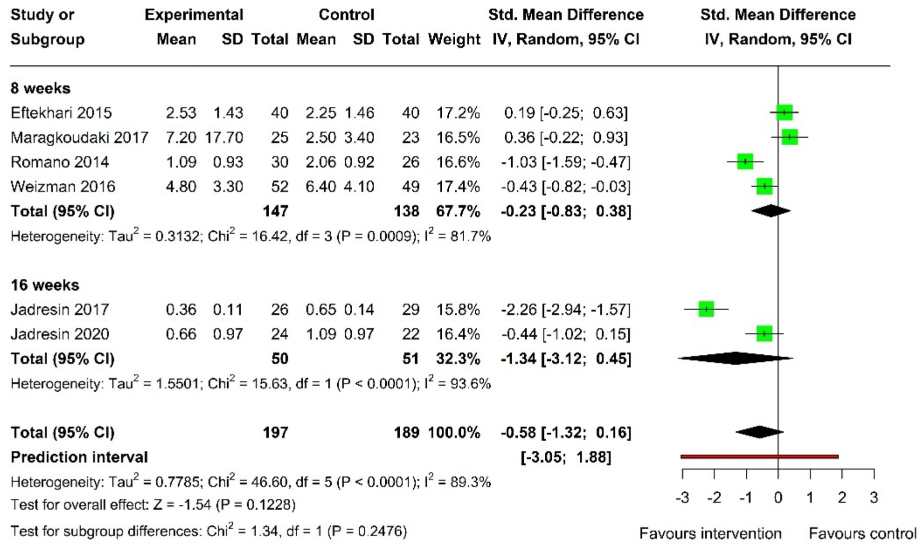

Figure S3. Pain severity at the end-of-follow-up *L. reuteri* DSM 17938 dose per day subgroup forest plot.

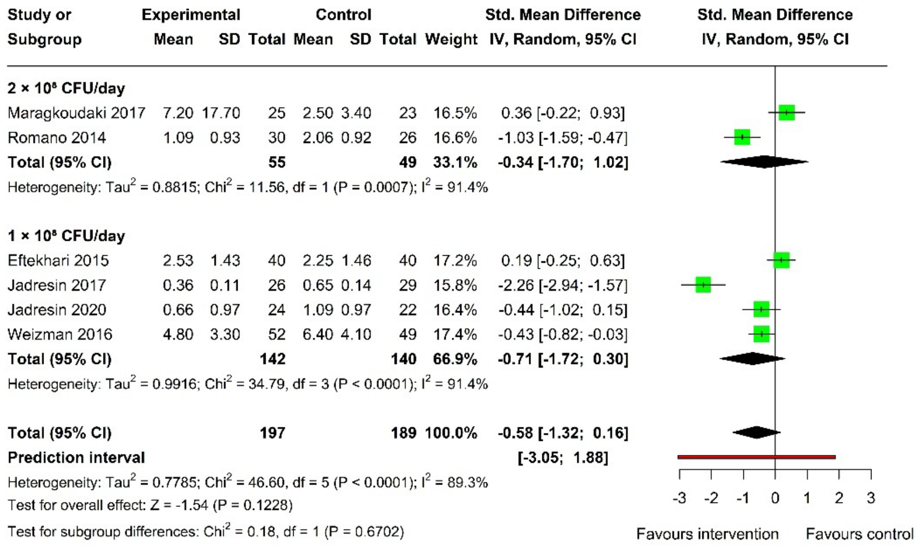

Figure S4. Pain severity at the end-of-follow-up *L. reuteri* DSM 17938 supplement presentation subgroup forest plot.

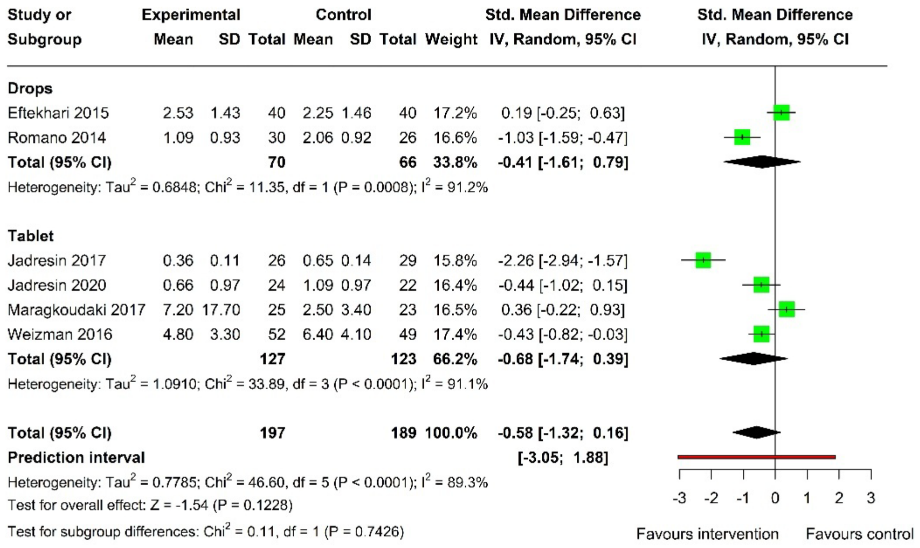

Figure S5. Pain frequency at the end-of-follow-up *L. reuteri* DSM 17938 dose per day subgroup forest plot.

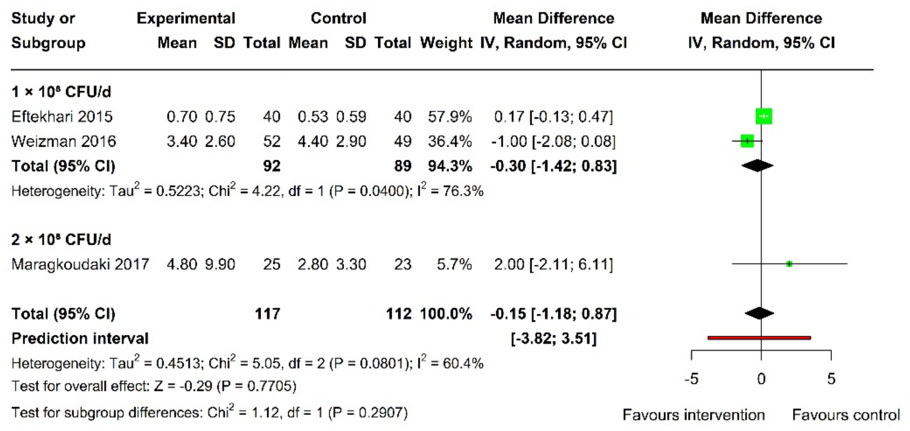

Figure S6. Pain frequency at the end-of-follow-up *L. reuteri* DSM 17938 supplement presentation subgroup forest plot.

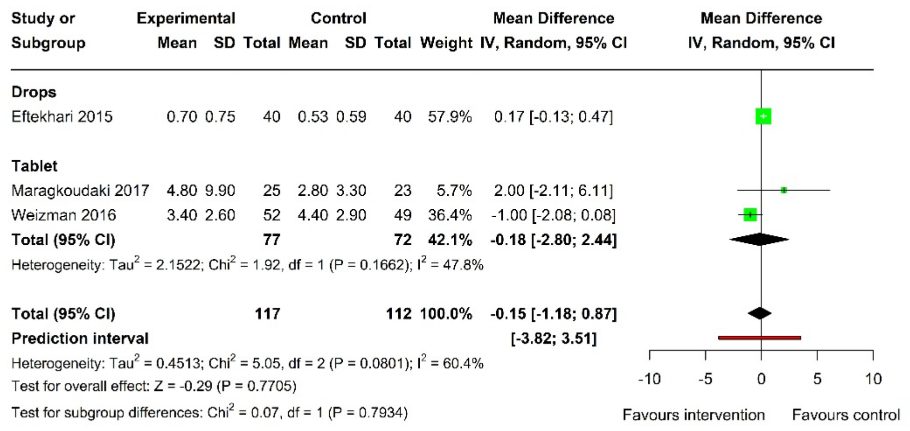

Figure S7. Use of drugs at the end-of-treatment primary meta-analysis with borrowed variance imputation.

Study	Experimental Mean	Experimental SD	Experimental Total	Control Mean	Control SD	Control Total	Weight	Mean Difference IV, Random, 95% CI
Maragkoudaki 2017	0.09	0.35	26	0.02	0.07	24	12.4%	0.07 [-0.07; 0.21]
Rahmani 2020	0.03	0.20	65	0.00	0.07	60	87.6%	0.03 [-0.02; 0.08]
**Random effects (Classic, 95% CI)**			**91**			**84**	**100.0%**	**0.03 [-0.01; 0.08]**
**Random effects (HKSJ, 95% CI)**								**0.03 [-0.13; 0.20]**
**Prediction interval**								**[-0.28; 0.35]**

Heterogeneity: $Tau^2 = 0$; $Chi^2 = 0.29$, df = 1 (P = 0.5934); $I^2 = 0.0\%$
Test for overall effect: Z = 1.42 (P = 0.1570)

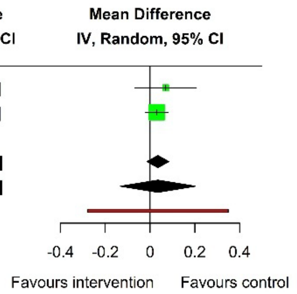

Note: For Rahmani 2020, SD was imputed from Maragkoudaki 2017 control group (SD = 0.07).

Figure S8. Use of drugs at the end-of-treatment sensitivity meta-analysis with near-zero variance imputation.

Study	Experimental Mean	Experimental SD	Experimental Total	Control Mean	Control SD	Control Total	Weight	Mean Difference IV, Random, 95% CI
Maragkoudaki 2017	0.09	0.35	26	0.02	0.07	24	11.1%	0.07 [-0.07; 0.21]
Rahmani 2020	0.03	0.20	65	0.00	0.00	60	88.9%	0.03 [-0.02; 0.08]
**Random effects (Classic, 95% CI)**			**91**			**84**	**100.0%**	**0.03 [-0.01; 0.08]**
**Random effects (HKSJ, 95% CI)**								**0.03 [-0.13; 0.19]**
**Prediction interval**								**[-0.26; 0.33]**

Heterogeneity: $Tau^2 = 0$; $Chi^2 = 0.29$, df = 1 (P = 0.5907); $I^2 = 0.0\%$
Test for overall effect: Z = 1.47 (P = 0.1407)

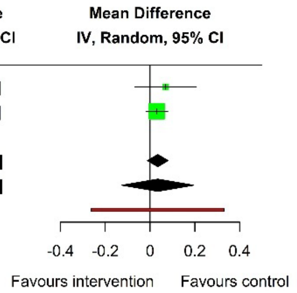

Note: For Rahmani 2020, SD was imputed as 0.0001 to approximate near-zero variance

Figure S9. Use of drugs at the end-of-treatment sensitivity meta-analysis with half of the borrowed variance imputation.

Study	Experimental Mean	Experimental SD	Experimental Total	Control Mean	Control SD	Control Total	Weight	Mean Difference IV, Random, 95% CI
Maragkoudaki 2017	0.09	0.35	26	0.02	0.07	24	11.5%	0.07 [-0.07; 0.21]
Rahmani 2020	0.03	0.20	65	0.00	0.04	60	88.5%	0.03 [-0.02; 0.08]
**Random effects (Classic, 95% CI)**			**91**			**84**	**100.0%**	**0.03 [-0.01; 0.08]**
**Random effects (HKSJ, 95% CI)**								**0.03 [-0.13; 0.20]**
**Prediction interval**								**[-0.27; 0.34]**

Heterogeneity: $Tau^2 = 0$; $Chi^2 = 0.29$, df = 1 (P = 0.5914); $I^2 = 0.0\%$
Test for overall effect: Z = 1.46 (P = 0.1450)

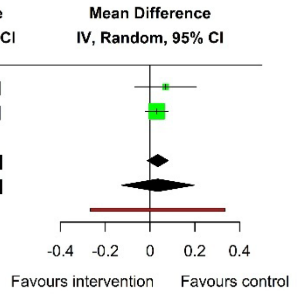

For Rahmani 2020, SD was imputed as 0.035 (50% of SD from Maragkoudaki 2017 control group)

Supplementary Table S1. Quantitative synthesis of rescue medication use (use of drugs) according to analytical scenario.

Analytical scenario	Studies included	SD imputation for Rahmani 2020	MD (days/week)	95% CI	I^2 (%)
No imputation	1	Not applicable (Rahmani excluded)	0.07	−0.07 to 0.21	—
Primary analysis (borrowed variance)	2	SD borrowed from the control group of Maragkoudaki 2017 (SD = 0.07)	0.03	−0.01 to 0.08	0
Sensitivity analysis 1 (near-zero variance)	2	SD = 0.0001	0.03	−0.01 to 0.08	0
Sensitivity analysis 2 (reduced borrowed variance)	2	SD = 0.035 (50% of borrowed SD from the control group of Maragkoudaki 2017)	0.03	−0.01 to 0.08	0

MD: mean difference; CI: confidence interval; SD: standard deviation. Rescue medication use was reported as days using medication per week. The primary quantitative synthesis used an SD borrowed from the control group of Maragkoudaki et al., 2017 (SD = 0.07) because Rahmani et al., 2020 reported a standard deviation of zero. Reduced borrowed and near-zero SD scenarios were conducted to evaluate the robustness of the pooled estimate under alternative assumptions regarding the missing variance.

Table S2. GRADE evidence profile of all outcomes.

Question: Lactobacillus Reuteri compared to Placebo for pediatric functional abdominal pain disorder

Certainty assessment							№ of patients		Effect	Certainty	Importance
**№ of studies**	**Study design**	**Risk of bias**	**Inconsistency**	**Indirectness**	**Imprecision**	**Other considerations**	**Lactobacillus Reuteri**	**Placebo**	**Absolute (95% CI)**		
**Pain severity at the end-of-follow-up (follow-up: range 8 weeks to 16 weeks; assessed with: Pain scales (Wong-Baker and Hicks's Faces Pain Scale Revised))**											
6	randomised trials	not serious[a]	very serious[b]	not serious	very serious[c]	none	197	189	SMD **0.58 SD lower** (1.32 lower to 0.16 higher)	⊕◯◯◯ Very low[a,b,c]	CRITICAL
**Pain severity at the end-of-treatment (follow-up: range 4 weeks to 16 weeks; assessed with: Pain scales (Wong-Baker and Hicks's Faces Pain Scale Revised))**											
7	randomised trials	not serious[d]	very serious[e]	not serious	serious[f]	none	263	250	SMD **0.49 SD lower** (0.93 lower to 0.06 lower)	⊕◯◯◯ Very low[d,e,f]	CRITICAL
**Pain frequency at the end-of-follow-up (follow-up: 8 weeks; assessed with: Number of episodes per week)**											
3[g]	randomised trials	not serious	serious[h]	not serious	very serious[i]	publication bias strongly suspected[j]	117[k]	112[l]	MD **0.15 episodes per week lower** (1.18 lower to 0.87 higher)	⊕◯◯◯ Very low[h,i,j]	IMPORTANT
**Pain frequency at the end-of-treatment (follow-up: 4 weeks; assessed with: Number of episodes per week)**											
3[g]	randomised trials	not serious	very serious[m]	not serious	very serious[i]	publication bias strongly suspected[j]	118[n]	113[o]	MD **0.6 episodes per week lower** (2.01 lower to 0.81 higher)	⊕◯◯◯ Very low[i,j,m]	IMPORTANT

Certainty assessment							№ of patients		Effect	Certainty	Importance
№ of studies	Study design	Risk of bias	Inconsistency	Indirectness	Imprecision	Other considerations	Lactobacillus Reuteri	Placebo	Absolute (95% CI)		
**Adverse events (follow-up: range 8 weeks to 16 weeks)**											
6	randomised trials	very serious[p]	not serious	not serious	not serious	none				⊕⊕◯◯ Low[p]	IMPORTANT
**School absenteeism (follow-up: range 4 weeks to 16 weeks)**											
5[q]	randomised trials	serious[r]	not serious	not serious	serious[s]	publication bias strongly suspected[t]	187	180	see comment	⊕◯◯◯ Very low[r,s,t]	IMPORTANT
**Work absenteeism at the end-of-follow-up (follow-up: 8 weeks; assessed with: Number of days lost of work per week)**											
1	randomised trials	not serious	not serious	not serious	very serious[s]	none	25	23	MD **0.12 days per week lower** (0.35 lower to 0.11 higher)	⊕⊕◯◯ Low[s]	NOT IMPORTANT
**Additional gastrointestinal symptoms frequency (follow-up: 8 weeks)**											
4[u]	randomised trials	serious[v]	not serious[w]	not serious	serious[s]	publication bias strongly suspected[x]	142	135	-	⊕◯◯◯ Very low[s,v,w,x]	IMPORTANT
**Additional gastrointestinal symptoms severity (follow-up: 8 weeks)**											
4[y]	randomised trials	serious[z]	not serious	not serious	serious[s]	publication bias strongly suspected[aa]	142	135	-	⊕◯◯◯ Very low[aa,s,z]	IMPORTANT

Certainty assessment							№ of patients		Effect	Certainty	Importance
**№ of studies**	**Study design**	**Risk of bias**	**Inconsistency**	**Indirectness**	**Imprecision**	**Other considerations**	**Lactobacillus Reuteri**	**Placebo**	**Absolute (95% CI)**		
**Healthcare utilization (as use of drugs) at the end-of-treatment (follow-up: range 4 weeks to 8 weeks; assessed with: days per week)**											
2[ab]	randomised trials	very serious[ac]	not serious	not serious	serious[s]	publication bias strongly suspected[ad]	91[ae]	84[af]	MD **0.03 days per week higher** (0.01 lower to 0.08 higher)	⊕◯◯◯ Very low[ac,ad,s]	IMPORTANT

Table S3. PRISMA 2020 Checklist.

Section and Topic	Item #	Checklist item	Location where item is reported
**TITLE**			
Title	1	Identify the report as a systematic review.	1
**ABSTRACT**			
Abstract	2	See the PRISMA 2020 for Abstracts checklist.	1
**INTRODUCTION**			
Rationale	3	Describe the rationale for the review in the context of existing knowledge.	2
Objectives	4	Provide an explicit statement of the objective(s) or question(s) the review addresses.	2-3
**METHODS**			
Eligibility criteria	5	Specify the inclusion and exclusion criteria for the review and how studies were grouped for the syntheses.	3
Information sources	6	Specify all databases, registers, websites, organizations, reference lists and other sources searched or consulted to identify studies. Specify the date when each source was last searched or consulted.	3-4
Search strategy	7	Present the full search strategies for all databases, registers and websites, including any filters and limits used.	3-4 and Supplementary Material
Selection process	8	Specify the methods used to decide whether a study met the inclusion criteria of the review, including how many reviewers screened each record and each report retrieved, whether they worked independently, and if applicable, details of automation tools used in the process.	4
Data collection process	9	Specify the methods used to collect data from reports, including how many reviewers collected data from each report, whether they worked independently, any processes for obtaining or confirming data from study investigators, and if applicable, details of automation tools used in the process.	4-5
Data items	10a	List and define all outcomes for which data were sought. Specify whether all results that were compatible with each outcome domain in each study were sought (e.g. for all measures, time points, analyses), and if not, the methods used to decide which results to collect.	3-4
	10b	List and define all other variables for which data were sought (e.g. participant and intervention characteristics, funding sources). Describe any assumptions made about any missing or unclear information.	4
Study risk of bias assessment	11	Specify the methods used to assess risk of bias in the included studies, including details of the tool(s) used, how many reviewers assessed each study and whether they worked independently, and if applicable, details of automation tools used in the process.	5
Effect measures	12	Specify for each outcome the effect measure(s) (e.g. risk ratio, mean difference) used in the synthesis or presentation of results.	4-5
Synthesis methods	13a	Describe the processes used to decide which studies were eligible for each synthesis (e.g. tabulating the study intervention characteristics and comparing against the planned groups for each synthesis (item #5)).	4-5

Section and Topic	Item #	Checklist item	Location where item is reported
	13b	Describe any methods required to prepare the data for presentation or synthesis, such as handling of missing summary statistics, or data conversions.	5-6
	13c	Describe any methods used to tabulate or visually display results of individual studies and syntheses.	6
	13d	Describe any methods used to synthesize results and provide a rationale for the choice(s). If meta-analysis was performed, describe the model(s), method(s) to identify the presence and extent of statistical heterogeneity, and software package(s) used.	5-6
	13e	Describe any methods used to explore possible causes of heterogeneity among study results (e.g. subgroup analysis, meta-regression).	5-6
	13f	Describe any sensitivity analyses conducted to assess robustness of the synthesized results.	5-6
Reporting bias assessment	14	Describe any methods used to assess risk of bias due to missing results in a synthesis (arising from reporting biases).	5-6
Certainty assessment	15	Describe any methods used to assess certainty (or confidence) in the body of evidence for an outcome.	6
**RESULTS**			
Study selection	16a	Describe the results of the search and selection process, from the number of records identified in the search to the number of studies included in the review, ideally using a flow diagram.	6-7
	16b	Cite studies that might appear to meet the inclusion criteria, but which were excluded, and explain why they were excluded.	7 and Supplementary Material
Study characteristics	17	Cite each included study and present its characteristics.	7
Risk of bias in studies	18	Present assessments of risk of bias for each included study.	12-13
Results of individual studies	19	For all outcomes, present, for each study: (a) summary statistics for each group (where appropriate) and (b) an effect estimate and its precision (e.g. confidence/credible interval), ideally using structured tables or plots.	12-13
Results of syntheses	20a	For each synthesis, briefly summarise the characteristics and risk of bias among contributing studies.	13
	20b	Present results of all statistical syntheses conducted. If meta-analysis was done, present for each the summary estimate and its precision (e.g. confidence/credible interval) and measures of statistical heterogeneity. If comparing groups, describe the direction of the effect.	13-17
	20c	Present results of all investigations of possible causes of heterogeneity among study results.	13-17
	20d	Present results of all sensitivity analyses conducted to assess the robustness of the synthesized results.	
Reporting biases	21	Present assessments of risk of bias due to missing results (arising from reporting biases) for each synthesis assessed.	17

Section and Topic	Item #	Checklist item	Location where item is reported
Certainty of evidence	22	Present assessments of certainty (or confidence) in the body of evidence for each outcome assessed.	19-22
**DISCUSSION**			
Discussion	23a	Provide a general interpretation of the results in the context of other evidence.	22-25
	23b	Discuss any limitations of the evidence included in the review.	25
	23c	Discuss any limitations of the review processes used.	25
	23d	Discuss implications of the results for practice, policy, and future research.	22-25
**OTHER INFORMATION**			
Registration and protocol	24a	Provide registration information for the review, including register name and registration number, or state that the review was not registered.	1
	24b	Indicate where the review protocol can be accessed, or state that a protocol was not prepared.	3
	24c	Describe and explain any amendments to information provided at registration or in the protocol.	NA
Support	25	Describe sources of financial or non-financial support for the review, and the role of the funders or sponsors in the review.	26
Competing interests	26	Declare any competing interests of review authors.	26
Availability of data, code and other materials	27	Report which of the following are publicly available and where they can be found: template data collection forms; data extracted from included studies; data used for all analyses; analytic code; any other materials used in the review.	26

From: Page MJ, McKenzie JE, Bossuyt PM, Boutron I, Hoffmann TC, Mulrow CD, et al. The PRISMA 2020 statement: an updated guideline for reporting systematic reviews. BMJ 2021;372:n71. doi: 10.1136/bmj.n71. This work is licensed under CC BY 4.0. To view a copy of this license, visit https://creativecommons.org/licenses/by/4.0/
